# Antileukemic Scalarane Sesterterpenoids and Meroditerpenoid from *Carteriospongia (Phyllospongia)* sp., Induce Apoptosis via Dual Inhibitory Effects on Topoisomerase II and Hsp90

**DOI:** 10.1038/srep36170

**Published:** 2016-10-31

**Authors:** Kuei-Hung Lai, Yi-Chang Liu, Jui-Hsin Su, Mohamed El-Shazly, Chih-Fung Wu, Ying-Chi Du, Yu-Ming Hsu, Juan-Cheng Yang, Ming-Kai Weng, Chia-Hua Chou, Guan-Yu Chen, Yu-Cheng Chen, Mei-Chin Lu

**Affiliations:** 1 Graduate Institute of Marine Biology, National Dong Hwa University, Pingtung, 944, Taiwan; 2 National Museum of Marine Biology & Aquarium, Pingtung 944, Taiwan; 3 Graduate Institute of Natural Products, College of Pharmacy, Kaohsiung Medical University, Kaohsiung 807, Taiwan; 4 Division of Pharmacognosy, Department of Medicinal Chemistry, Uppsala University, Uppsala, Sweden; 5 Division of Hematology-Oncology, Department of Internal Medicine, Kaohsiung Medical University Hospital, Kaohsiung, 807, Taiwan; 6 Department of Internal Medicine, Faculty of Medicine, College of Medicine, Kaohsiung Medical University, Kaohsiung, 807, Taiwan; 7 Department of Pharmacognosy and Natural Products Chemistry, Faculty of Pharmacy, Ain-Shams University, Organization of African Unity Street, Abassia, Cairo 11566, Egypt; 8 Division of Surgical Oncology, Department of Surgery, Kaohsiung Medical University Hospital, Kaohsiung 807, Taiwan; 9 School of Pharmacy, College of Pharmacy, China Medical University, Taichung, Taiwan; 10 Chinese Medicine Research and Development Center, China Medical University Hospital, Taichung, Taiwan; 11 The Ph.D. Program for Cancer Biology and Drug Discovery, China Medical University and Academia Sinica, Taichung, Taiwan

## Abstract

Two new scalarane sesterterpenoids, 12*β*-(3′*β*-hydroxybutanoyloxy)-20,24-dimethyl-24-oxo-scalara-16-en-25-al (**1**) and 12*β*-(3′*β*-hydroxypentanoyloxy)-20,24-dimethyl-24-oxo-scalara-16-en-25-al (**2**), along with one known tetraprenyltoluquinol-related metabolite (**3**), were isolated from the sponge *Carteriospongia* sp. In leukemia Molt 4 cells, **1** at 0.0625 μg/mL (125 nM) triggered mitochondrial membrane potential (MMP) disruption and apoptosis showing more potent effect than **2** and **3**. The isolates inhibited topoisomerase IIα expression. The apoptotic-inducing effect of **3** was supported by the *in vivo* experiment through suppressing the volume of xenograft tumor growth (47.58%) compared with the control. Compound **1** apoptotic mechanism of action in Molt 4 cells was further elucidated through inducing ROS generation, calcium release and ER stress. Using the molecular docking analysis, **1** exhibited more binding affinity to N-terminal ATP-binding pocket of Hsp90 protein than 17-AAG, a standard Hsp90 inhibitor. The expression of Hsp90 client proteins, Akt, p70^S6k^, NFκB, Raf-1, p-GSK3β, and XIAP, MDM 2 and Rb2, and CDK4 and Cyclin D3, HIF 1 and HSF1 were suppressed by the use of **1**. However, the expression of Hsp70, acetylated tubulin, and activated caspase 3 were induced after **1** treatment. Our results suggested that the proapoptotic effect of the isolates is mediated through the inhibition of Hsp90 and topoisomerase activities.

Heat shock proteins (Hsp) comprise a certain group of highly conserved stress proteins which attracted attention due their overexpression in cancer tissues^1^,^2^. Overexpression of these proteins is related to metastatic potential, resistance to chemotherapy and poor prognosis^3^. They are named depending on their molecular weight (Hsp60, Hsp70 and Hsp90) and among the most studied protein is Hsp90. This protein is the most prominent member of the highly abundant chaperone proteins and it is essential for folding nascent polypeptide to control the activity, stability and protein sorting^4^. Hsp90 has been identified as a promising drug target for cancer treatment, because it can stabilize and activate a variety of survival proteins to maintain cancer phenotype and help cancer cells to overcome multiple environmental stresses^5^. There is considerable interest in developing potential Hsp90 inhibitors, with a much simpler rationale, through the depletion of oncogenic Hsp90 clients^6^. The structure of this protein is composed of three major regions: an amino (N)-terminal domain with an adenosine triphosphate (ATP)-binding and hydrolyzing pocket (with ATPase activity), a middle domain involved in client protein recognition/binding, and a carboxy (C)-terminal domain^7^. Tanespimycin (17-allylamine-17-demethoxygeldanamycin, 17-AAG), the first Hsp90 inhibitor, was found to bind to the N-terminal regulatory pocket of Hsp90 and thus inhibiting its function. In Phase I clinical trials, it showed promising anticancer activity against multiple myeloma in combination with bortezomib^8^,^9^. However, further clinical development of 17-AAG was halted in 2010 because of poor solubility, limited bioavailability, unaccepted hepatotoxicity and the most important factor was the expiration of the patent in 2014^4^,^10^,^11^. To date, several Hsp90 inhibitors have entered clinical trials, but none of them has been approved as an anticancer agent^12^.

Another important group of proteins, topoisomerases (Topo), has also attracted attention due to their crucial role in cell survival and replication^13^. Topoisomerases are classified into two main classes: topoisomerase I and topoisomerase II with each class performing specific functions in the cell. Topoisomerase II is composed of two isoforms, α and β, which share highly similar amino acid sequence (up to 70%)^14^. Topo IIα is essential for the survival of proliferating cells and can distinguish the handedness of DNA supercoils during relaxation reactions; however, Topo IIβ is dispensable at the cellular level^15^,^16^. Topo II disentangle topological problems, which regulate DNA replication, transcription and chromosome segregation, as well as processes related to tumorigenesis^17^. Inhibition of Topo II activity is one of the current therapeutic protocols targeting several cancers including lung, breast, lymphomas, testicular and sarcomas^18^.

The inhibition of Topo II activity is achieved either with poisons, which interfere with the topoisomerase-DNA complex or inhibitors, which suppress the catalytic turnover. Topo II poisons are divided into two distinct classes, interfacial poison and covalent poison based on their mechanism of action^15^,^19^. Anticancer drugs such as etoposide, doxorubicin, mitoxantrone and bioflavonoid analogs are considered interfacial poisons that bind non-covalently to the cleavage complex at the protein-DNA interface^15^,^17^. On the other hand, epigallocatechin galate (EGCG) and curcumin are covalent poisons which function distal to the active site of Topo II and increase the level of enzyme-mediated DNA cleavage by altering conformation of Topo II N-terminal protein gate^20^,^21^,^22^. Despite the effectiveness of Topo II poisons as anticancer drugs, they can trigger chromosomal breaks leading to secondary leukemogenesis^14^,^23^,^24^. One solution of this side effect was the development of catalytic Topo II inhibitors such as bisdioxopiperazines which eliminate the essential enzymatic activity of Topo IIα^17^. These compounds showed modest anticancer activity but did not induce secondary malignancies. Further developments of catalytic Topo II inhibitors can introduce new classes of highly effective and relatively safe anticancer agents.

Natural products scaffolds have played a crucial rule in guiding researchers to develop efficient anticancer agents targeting proteins necessary for cancer cell survival and replication^11^. Certain classes of secondary metabolites exhibited potent anticancer activity such as terpenoids, alkaloids, and flavonoids^25^. Terpenoids are the largest and most diverse group of secondary metabolites which are divided into several subgroups including monoterpenoids, diterpenoids, sesquiterpenoids, sesterterpenes, and triterpenoids^26^. Scalarane sesterterpenoids emerged as an interesting group of terpenoids which were isolated from marine sponges and shell-less mollusks. Scalarane-type sesterpenoids are 25C-terpenoids with tetra- or penta-cyclic skeletons derived from scalarin, which was firstly isolated from the sponge *Cacospongia scalaris* in 1972^27^. Scalarane sesterterpenoids displayed a wide spectrum of interesting biological properties, such as antifeedant, antimicrobial, antifungal, ichthyotoxicity, antitubercular, antitumor, cytotoxicity, anti-HIV, antifouling, inhibition of platelet-aggregation, inhibition of transactivation for the nuclear hormone receptor (FXR, farnesoid X-activated receptor), stimulation of nerve growth factor synthesis, as well as anti-inflammatory activity^28^. Recent reports from our group revealed the potent cytotoxic potential of scalarane-type sesterterpenoids^29^,^30^. *Carteriospongia* (*Phyllospongia*) sp. sponge is a rich source of bioactive scalarane sesterterpenoids^31^. Unfortunately, many of the cytotoxic studies on sesterterpenoids evaluated their cytotoxic effect on cancer cell lines without deviling deeper into their mechanism of action^32^. Relieving the cytotoxic mechanism of action is crucial for discovering cytotoxic drug leads and fine tuning the active compounds to improve potency and reduce cytotoxicity. In the current investigation, we examined the content of EtOAc extract of *Carteriospongia* (*Phyllospongia*) sp. which led to the isolation of two new scalarane sesterterpenoids and one known tetraprenyltoluquinol-related metabolite. We evaluated the cytotoxic activity of isolates *in vitro* and *in vivo* along with their mechanism of action. The isolates caused disruption in mitochondrial membrane potential suggesting that the apoptotic effect is mediated through mitochondrial dysfunction. The cytotoxic effect of the new scalarane-type sesterterpenoid (**1**) on human leukemia Molt 4 cells involved induction of ER stress as demonstrated by the increase in ROS generation as well as ATF 6 cleavage and Chop expression. Compound **1** exhibited potent Hsp90 and Topoisomerase II (Topo II) catalytic inhibitory activity as determined by molecular docking and cell-free system. The treatment of Molt 4 cells with compound **1** suppressed client protein expression and accumulation of Hsp70 in the cytosolic compartment and well as reduced the transcription factors (HSF 1 and HIF 1) expression as demonstrated by Western blot and immunofluorescent analyses.

## Results

### Chemical identification of these marine terpenoids

**Figure 1:**
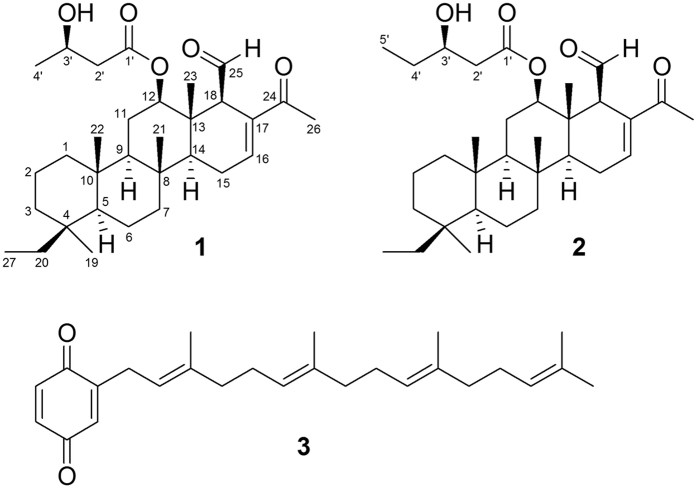
Terpenoids from *Carteriospongia (Phyllospongia)* sp.

The EtOAc extract of the freeze-dried specimen was fractionated by silica gel column chromatography and the eluted fractions were further separated utilizing normal phase HPLC to yield **1**–**3** (Fig. 1). The new compounds were named as 12*β*-(3′*β*-hydroxybutanoyloxy)-20,24-dimethyl-24-oxo-scalara-16-en-25-al (**1**) and 12*β*-(3′*β*-hydroxy-pentanoyloxy)-20,24-dimethyl-24-oxo-scalara-16-en-25-al (**2**). The known compound was identified as 2-tetraprenil-1,4-benzochinone (**3**)^33^.

**Figure 2:**
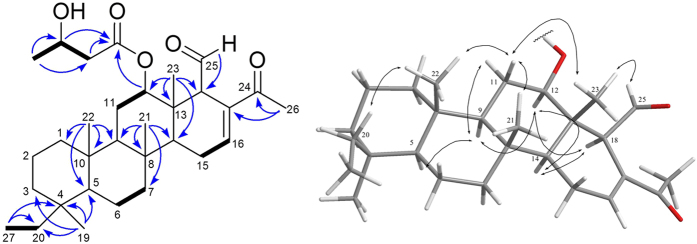
Selected ^1^H–^1^ H COSY (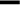), HMBC (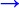) and NOESY (↔) correlations of 1.

Compound **1** was obtained as colorless oil. The molecular formula of **1** was determined to be C_31_H_48_O_6_ by HR-ESI-MS (*m/z* 523.3397 [M+Na]^+^) and ^13^C NMR data (Table 1), implying eight degrees of unsaturation. IR absorptions were observed at 3448, 1717 and 1665 cm^−1^, suggesting the presence of hydroxyl, saturated carbonyl and *α*,*β*-unsaturated carbonyl functionalities. Resonances due to an aldehyde carbonyl carbon (*δ*_C_ 201.1), *α*,*β*-unsaturated carbonyl carbon (*δ*_C_ 198.2), ester carbonyl carbon (*δ*_C_ 172.1) and olefinic carbons (*δ*_C_ 142.9, CH; 138.4, C) in the ^13^C NMR and DEPT spectral data accounted for four double-bond equivalents, indicating a tetracyclic skeleton of **1**. In the ^1^H NMR data, resonances of one olefinic proton (*δ*_H_ 7.06, s) and two oxygenated methines (*δ*_H_ 4.86, dd, *J* = 11.0, 4.5 Hz; 4.22, t, *J* = 9.0 Hz) were observed. The planar structure and all of the ^1^H and ^13^C chemical shifts of **1** were elucidated by 2D NMR spectroscopic analysis, in particular ^1^H-^1^H COSY and HMBC experiments (Fig. 2), suggesting a characteristic scalarin-type sesterterpenoid structure. Compound **1** was found to possess a 3-hydroxybutanoyloxy at C-12, a double bond at C-16/C-17, a ketone group at C-24 and an aldehyde group at C-25. Moreover, the structure of **1** was established unambiguously, and was found to be similar to that of a known compound, dendalone 3-hydroxybutyrate^8^,^34^. The relative configuration of **1** was suggested to be similar to that of dendalone 3-hydroxybutyrate by comparison of the chemical shifts and coupling constants of both compounds protons and was further confirmed by NOESY correlations (Fig. 2). The 3′*R*-configuration was determined by comparing the optical rotation (+10.7) with those of **2** (+5.7). The structure of **1** was thus found to possess the (4S*, 5S*, 8R*, 9R*, 10S*, 12R*, 13S*, 14S*, 18R*, 3′R*)-configuration.

Compound **2** molecular formula was deduced as C_32_H_50_O_5_ based on HR-ESI-MS (ion peak at *m/z* 537.3546 [M+Na]^+^) and ^13^C NMR data suggesting that **2** possesses one more CH_2_ group compared with **1**. The NMR data of **2** (Table 1) showed similarity to those of **1** with the replacement of one 3-hydroxybutanoyloxy group at C-12 in **1** by one 3-hydropentanolyoxy group in **2**. The structure of compound **2** was further confirmed by the COSY correlations from H_2_-29 to H_3_-32 and by the HMBC correlations from H-12 and H_2_-29 to C-28. Both compounds (**1** and **2**) were suggested to possess identical relative configuration based on the similarity in their NMR data. In order to identify the C-3′ configuration on the 3-hydroxybutanoyloxy group, the (*S*)- and (*R*)-MTPA ester derivatives, **2s** and **2r**, were synthesized with (−)-(*R*)-MTPA-Cl and (+)-(*S*)-MTPA-Cl, respectively. The Δ*δ*-values indicated 3′*R*-configuration (Figure S19).

### Apoptotic induction of these marine terpenoids via DNA damage and MMP dysfunction

**Figure 3:**
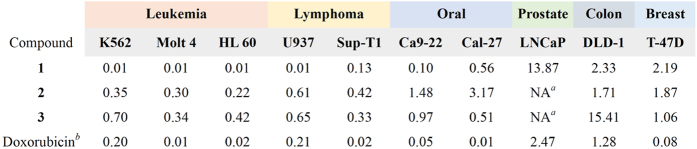
Cytotoxic effects of 1–3 against several cancer cell lines for 72 h (IC_50_, μg/mL). ^a^NA (non-active) = IC_50_ > 20 *μ*g/mL for 72 h. ^b^Positive control.

**Figure 4:**
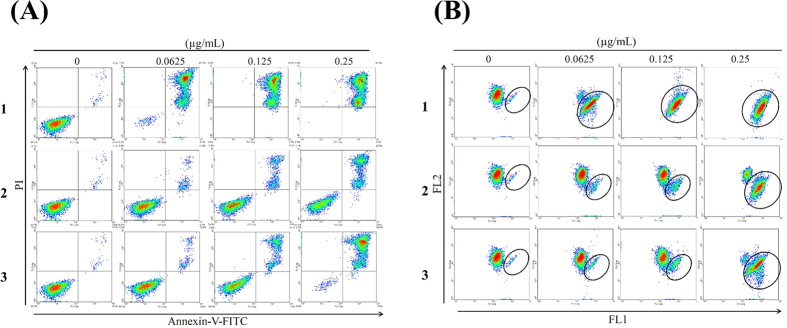
Effect of compounds 1–3 on apoptosis induction and MMP disruption. Cells were treated with the indicated concentration of **1**–**3** for 24 h, respectively. (**A**) Apoptosis induction and (**B**) mitochondrial membrane potential were assessed with annexin V/PI and JC-1 staining using flow cytometric analysis.

The antiproliferative effect of the two new scalarane sesterterpenoids and the known tetraprenyltoluquinol-related metabolite was evaluated using MTT assay. Several cancer cell lines including leukemia K562, Molt 4, and HL 60 cells, prostate cancer LNCaP cells, colon cancer DLD-1 cells and breast cancer T-47D cells were used to evaluate the antiproliferative activity. Figure 3 summarized the data of IC_50_ values in cytotoxicity of these marine natural products against several cancer cell lines. Compound **1** exhibited the most potent cytotoxic activity with an IC_50_ of 0.01 μg/mL (2.08 nM) against all leukemia and one lymphoma cell lines. After 72 h treatment, the IC_50_ values of **1** against DLD-1, T-47D, LNCaP, Ca9-22 and Cal-27 cells were 2.33, 2.19, 13.87, 0.1, and 0.56 μg/mL, respectively. The IC_50_ values of **2** against leukemia K562, Molt 4 and HL 60 cells were 0.35 and 0.30, 0.22 μg/mL which were comparable to those of **3** (IC_50_ values: 0.70, 0.34 and 0.42 μg/mL). Molt 4 cell line was the most sensitive cell line as demonstrated by MTT assay. To detect whether the cytotoxic effect of these terpenoids was associated with the mitochondria-related apoptosis, we assessed the population of apoptosis and disruption of mitochondrial membrane potential in Molt 4 cells with annexin-V/PI and JC-1 staining. After 24 h, compounds **1**, **2** and **3** resulted in a dose-dependent (0, 0.0625, 0.125 and 0.25 μg/mL) increase in the apoptotic population of Molt 4 cells and disruption in mitochondrial membrane potential (Fig. 4A,B). These results proved that the cytotoxic activity of these marine terpenoids might be mediated through mitochondrial dysfunction leading to induction of apoptosis.

**Figure 5:**
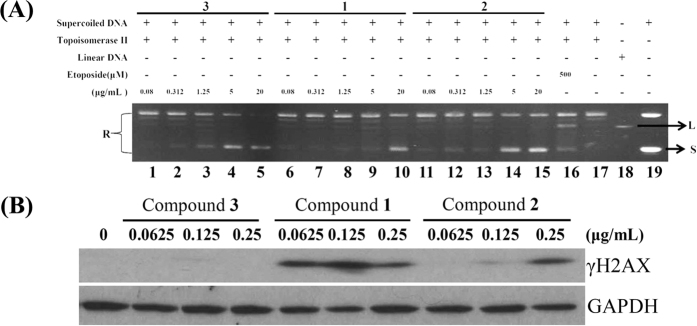
Effect of marine terpenoids on Topo II α activity. (**A**) Effect of compounds **1**–**3** on topo II activity. Lanes 1–5: **3** (0.08, 0.3125, 1.25, 5, and 20 μg/mL); Lanes 6–10: **1** (0.08, 0.3125, 1.25, 5, and 20 μg/mL); Lanes 11–15: **2** (0.08, 0.3125, 1.25, 5, and 20 μg/mL); Lane 16: positive control, etoposide (500 μM), as topo II poison (induction of linear DNA); Lane 17: plasmid DNA + topo II + solvent control (induction of DNA relaxation); Lane 18: Linear DNA; Lane 19: negative control plasmid DNA (supercoiled DNA); The full length gel of Topo IIα is supplied in Supplementary data Fig. S20 (**B**) The treatment with marine terpenoids induced the expression of γH2AX protein in Molt 4 cells. The cells were treated with compounds **1**-**3** (0, 0.0625, 0.125 and 0.25 μg/mL) for 24 h, respectively. Protein expression was analyzed with Western blotting. GAPDH was used the loading control.

The ability of Topo II to generate DNA damage in the presence of Topo II-targeting agents led to the hypothesis that an important determinant of drug sensitivity was related to the overall level of Topo II^17^. In our previous report, 10-acetylirciformonin B, a marine sesterterpenoid analog, significantly inhibited Topo IIα activity^35^. Such findings encouraged us to evaluate the effect of the isolates on Topo II activity. In order to confirm whether the DNA damage-induced by the isolated sesterterpenoids involved the inhibition of Topo II activity, a cell-free DNA cleavage assay using an enzyme-mediated negatively supercoiled pHOT1 plasmid DNA was applied. A linear DNA strand was observed upon treating the supercoiled pHOT1 plasmid DNA with etoposide, a standard topo II poison (Lane 16). The use of compound **1** in increasing concentrations (0.08, 0.312, 1.25, 5, and 20 μg/mL) significantly inhibited DNA relaxation by 12, 13, 17, 20 and 99%, respectively, compared with the control supercoiled DNA and resulted in the formation of supercoiled DNA products in the presence of topo IIα (Lanes 6–10). Additionally, **2** and **3** significantly inhibited DNA relaxation by 12, 23, 36, 90 and 99% (Lanes 11–15); and 7, 13, 28, 90 and 98% (Lanes 1–5), respectively. These marine terpenoids suppressed Topo II activity, resulting in the inhibition of supercoiled DNA relaxation in a dose-dependent manner (Fig. 5A). Furthermore, **1**-**3** inhibited topo II activities with IC_50_ of 1.98, 0.37 and 0.43 μg/mL, respectively as demonstrated by the cell-free system. To determine whether the apoptotic mechanism of these compounds affects γH2AX (as a biomarker of DNA damage) induction, Western blot analysis was employed to examine the activation of γH2AX. Compounds **1** and **2** significantly enhanced the expression of γH2AX in a dose-dependent manner, but the activation of DNA damage was not observed on **3** (Fig. 5B). These results suggested that these compounds could act as potent catalytic inhibitors of Topo IIα.

### 2-Tetraprenil-1,4-benzochinone (3) inhibited tumor growth in *in vivo* human Molt 4 tumor xenograft animal model

**Figure 6:**
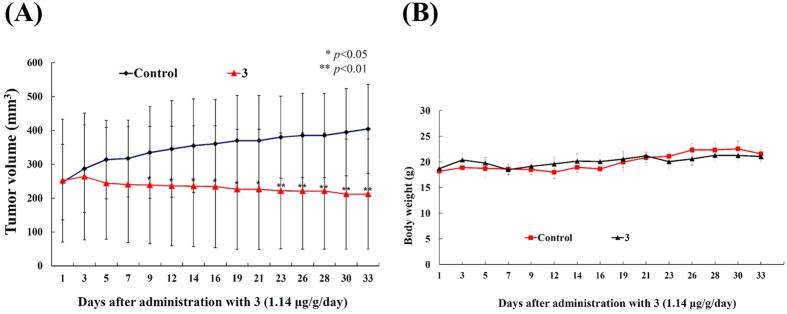
Effect of compound 3 on tumor growth and body weight in *in vivo* human Molt 4 tumor xenograft animal model. Tumor-bearing nude mice were intraperitoneally injected with solvent control (DMSO) and **3** (1.14 μg/g) for 33 days. (**A**) Tumor volumes were measured every other day, and the results are expressed as mean ± SD. *Significantly different from control groups at *p < 0.05; **p < 0.01. (**B**) The body weight were measured every other day, and the results are expressed as mean ± SD. Control, n = 8; Compound **3**, n = 7.

Compounds **1** and **2** induced apoptosis in several cancer cell lines; however, the amounts isolated were insufficient for further examination of their *in vivo* antitumor effect. On the other hand, the amount separated from **3** was sufficient to study the *in vivo* anti-tumor activity by evaluating its effect on tumor growth of human leukemia Molt 4 in xenograft animal model. Molt 4 (1 × 10^6^) cells were inoculated subcutaneously at the right flank of female immunodeficient athymic mice. After 33-day of treatment, the tumor growth of Molt 4 cells was significantly suppressed under the influence of **3** (1.14 μg/g) intraperitoneal injection. The average tumor size on day 33 in the control group was 404.63 mm^3^, whereas the average tumor size in compound **3**-treated group was 212.10 mm^3^ (Fig. 6A). The tumor size was significantly lower in compound **3**-treated group as compared to the control group (*p* < 0.05) with no significant difference in the mice body weights (Fig. 6B). These results suggested that **3** exhibited anti-tumorigenic effect *in vivo* xenograft model.

### Apoptotic effect of 12*β*-(3′*β*-hydroxybutanoyloxy)-20,24-dimethyl-24-oxo-scalara-16-en-25-al (1) involves the induction of ROS generation, ER stress and DNA damage in Molt 4 cells

**Figure 7:**
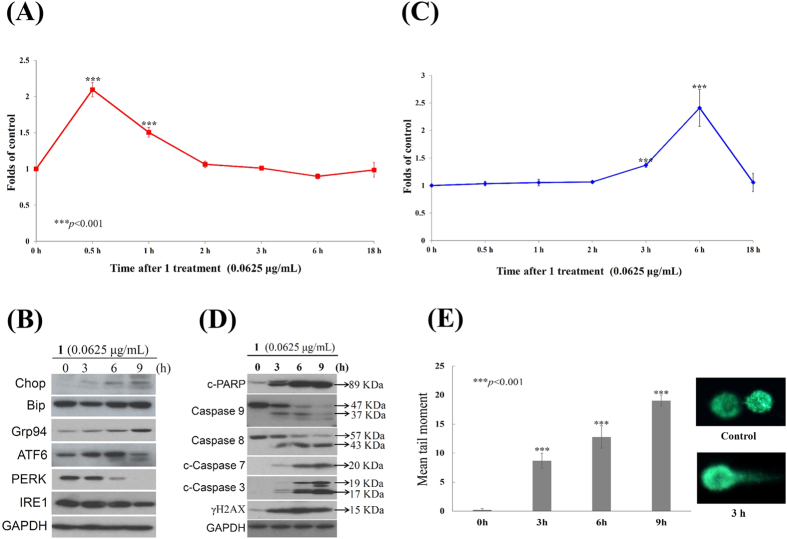
Apoptotic effect of compound 1 involved the induction of ROS generation, ER stress and DNA damage in Molt 4 cells. (**A,C**) Effect of **1** on ROS generation and calcium accumulation. Cells were treated with **1** (0.0625 μg/mL) for the indicated times. Quantitative results showed a gradual increase in the ROS production or calcium accumulation in response to the **1** treatment when compared with the control group. (***p < 0.001); (**B,D**) Cells were harvested and lysates were prepared and subjected to SDS-PAGE followed by immunoblotting for ER- or apoptosis-related proteins. GAPDH was used as the loading control. The full length blots of caspase 9, 8 and 3 expression are supplied in Supplementary data Fig. S21 (**E**) An example of “comet tail” due to chromosomal DNA double-strand breaks in **1** (0.0625 μg/mL)-treated Molt 4 cells compared to the untreated control. Electrophoresis was carried out under neutral conditions. Quantitative results showed a gradual increase in tail movement upon **1** treatment for indicated time when compared with the control. Results are presented as mean ± SD of three independent experiments (*p < 0.05).

Oxidative stress is manifested by ROS overexpression and cannot be balanced by the available antioxidant machinery^36^. Compound **1** can damage the integrity of mitochondria, which are the major production sites of the superoxide anion, ozone^37^. The induction of the intracellular formation of ROS by **1** was determined with a carboxyl derivative of fluorescein, carboxy-H_2_DCFDA dye using flow cytometric analysis^38^. As shown in Fig. 7A, treatment with **1** (0.0625 μg/mL) for 0.5, 1, 2 and 3 h resulted in 2.09-, 1.51-, 1.06- and 1.01-fold increase in ROS levels, respectively, as compared with the mean fluorescence index (MFI) of the control. In addition, ROS generation could induce ER stress leading to mitochondria-related apoptosis^39^,^40^. To further investigate if ER stress is involved in the apoptotic effect induced by **1**, Western blot analysis was used to determine the expression of ER stress-related proteins. In a time-dependent manner, **1** promoted the levels of Bip, Chop, Grp 94 as well as the activation and cleavage of ATF 6 but suppressed the levels of PERK and IRE 1 (Fig. 7B). Furthermore, the effect of **1** on the release of intracellular Ca^2+^, was evaluated using fluorescent calcium indicator, Fluo 3. The flow cytometric results showed that the treatment with **1** at different time intervals (0.5, 1, 2, 3, 6 and 18 h) induced 1.03-, 1.05-, 1.06-, 1.37-, 2.41-, and 1.06-folds increase in the intracellular Ca^2+^ accumulation, respectively as compared to MFI of the control (Fig. 7C), indicating that ER stress was induced by compound **1** following the redox stress. Moreover, we noticed a significant increase of PARP, caspases-8 and −9 cleavages in a time-dependent manner. The induction of the typical executor caspases-3 and −7, as well as γH2AX, was also observed using Western blot analysis (Fig. 7D). To confirm the induction of DNA damage by **1** in Molt 4 cells, a comet assay under neutral electrophoresis condition was utilized. The effect of **1** (0.0625 μg/mL) at different time intervals (0, 3, 6 and 9 h) on the level of nuclear DNA integrity was determined. As shown in Fig. 7E 1 increased the degree of DNA migration in Molt 4 cells. The DNA migration was represented by the induction of DSBs in a time-dependent increase, as indicated by the abnormal tails’ sizes in the comet assay.

### 12*β*-(3′*β*-Hydroxybutanoyloxy)-20,24-dimethyl-24-oxo-scalara-16-en-25-al (1) as a potent inhibitor of Hsp90

**Figure 8:**
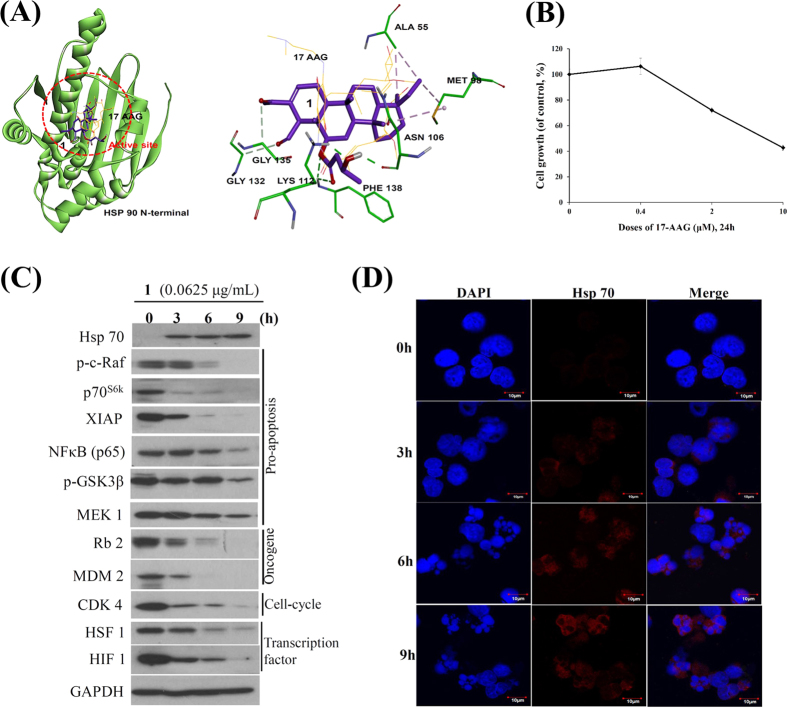
Compound 1 as the potent inhibitor of Hsp90. (**A**) Molecular modeling of Hsp90 protein with compound **1** as assessed by Autodock 4.2 software with Lamarckian Genetic Algorithm. (17-AAG: labeled with yellow; Compound **1**: labeled with purple). (**B**) Effect of 17-AAG on cytotoxicity of Molt 4 cells. (**C**) Effect of compound **1** on expression of Hsp90 client proteins. (**D**) Effect of compound **1** on localization of Hsp70 protein.

It is known that mitochondria are critical intracellular loci of ROS production and ROS exposure can lead to the mPTP opening^41^. The antioxidant, microsomal GST1, in the inner mitochondrial membrane can interact with the mitochondrial permeability transition (MPT) regulator proteins, such as ANT and/or CypD, to form a MPT pore contributing to mitochondria-mediated cell death^42^. It was reported that mitochondrial homeostasis of tumor cells is regulated by an organelle-specific Hsp90 chaperone network^43^. To investigate whether the inhibition of Hsp90 participates in the apoptosis induced by **1**, we performed molecular docking of Hsp90 with the available crystal structure to gain insight of **1** binding mode. Our studies revealed that **1** could be docked to the N-terminal domain of Hsp90 with the binding energy of −10.93 Kcal/mol over the first Hsp90 N-terminal inhibitor, 17-AAG (−6.63 Kcal/mol), presumably because **1** formed non-classic hydrogen bonds with GLY132 and GLY135 at a distance of 3.27 and 3.66 Å; the classic hydrogen bonds with residues LYS112 (1.92 Å), PHE138 (2.31 Å and 2.45 Å) and ASN106 (2.45 Å); the hydrophobic bonds with residues ALA 55 (3.63 and 3.69 Å) and MET 98 (3.85 and 4.96 Å) (Fig. 8A). According to the molecular docking experiments, compound **1** inhibits Hsp90 with an inhibition constant of 9.67 nM (>1430 folds) which was more potent than 17-AAG (10.83 μM). To fully understand the differences in activity between **1** and 17-AAG, we further examined the antiproliferative activity of 17-AAG in Molt 4 cells by MTT assay. Treatment of Molt 4 with increasing concentrations (0, 0.4, 2 and 10 μM of 17-AAG for 24 h induced a suppression in cell growth with IC_50_ values of 4.2 μg/mL (7.2 μM). (Fig. 8B). According to the MTT results using Molt 4 cells, the antiproliferative activity of **1** (0.01 μg/mL, 19.1 nM) was 377 folds higher than that of 17-AAG.

Recent reports indicated that Hsp90 inhibitors induced HSF 1-dependent expression of Hsp70^44^, and the genetic deletion of HSF 1 reduced the association of Hsp90 with its kinase client proteins^45^,^46^. It was also reported that the induction of Hsp70 is the biomarker of Hsp90 with N-terminal inhibition^3^,^6^,^7^. Aiming to understand the relation between Hsp90 function and the expression of Hsp70 and 90 clients, we treated Molt 4 cells with **1** (0.0625 μg/mL) for different time intervals. We first observed that **1** treatment did not notably attenuate the expression of Hsp90 protein. In agreement with earlier studies^47^,^48^,^49^, the expression of Hsp70 increased in a time-dependent manner, as an established marker for HSR after Hsp90 inhibition, while surprisingly the expression of HSF 1 protein was attenuated with **1** treatment in Molt 4 cells. As expected, the suppression of Hsp90 client proteins was observed, including p70^S6k^, NFκB, Raf-1, p-GSK3β, MEK 1 and XIAP (pro-apoptotic protein), MDM 2 and Rb2 (oncoprotein), and CDK4 (cell cycle regulatory protein), HIF 1 and HSF1 (transcription factor) (Fig. 8C). The majority of Hsps accumulate in subcellular localizations that determine whether a cell is going to die or differentiate. Erythroblasts with accumulated nuclear Hsp70 continued their differentiation process during the formation of red blood cells^50^. Furthermore, we identified the localization of Hsp70 in response to **1** treatment using immunofluorescence by confocal microscope. In agreement with the Western blot results, the localization of Hsp70 was predominantly accumulated in cytosol and the accumulation increased with time (Fig. 8D).

## Discussion

Clinically, there is an unmet medical need in the treatment of T-cell lymphoblastic leukemia. In comparison with other human malignancies such as colon cancer, breast cancer, prostate cancer where there has been much progress in their treatment strategies including molecular targeting therapy; patients with T-cell lymphoblastic leukemia are usually treated with conventional chemotherapy which is often associated with acute or chronic toxicities as well as a high relapse rate^51^,^52^. The outcome of refractory or relapsed disease remains poor and the search for more effective drugs or potential targets is urgently required. T-cell lymphoblastic leukemia Molt 4 cell line was the most sensitive cell line to treatment with the new isolated scalarane sesterterpenoids as determined with MTT assay (Fig. 3), reflecting their potential as novel anti-leukemic agents.

In this study, we investigated the therapeutic potential of scalarane sesterterpenoids (**1** and **2**) and a meroditerpenoid (**3**) in Molt 4 cell line. All compounds reduced cells viability and Topoisomerase II catalytic activity with comparable IC_50_ values. Flow cytometric analysis showed that **1** induced mitochondrial dysfunction and apoptotic cell death with the lowest dose (0.0625 μg/mL) after 24 h (Fig. 4A,B). Therefore, **1** was selected for further in depth studies to understand its apoptotic mechanism of action.

It was reported that Hsp90 facilitates the proper folding of signaling proteins associated with cancer progression, tumor angiogenesis and therapy resistance by functioning as molecular chaperone, gaining attention as a target for therapeutic intervention^47^,^48^,^53^. A growing body of evidence indicates that the accumulation of unfolding/misfolding proteins by the suppression of Hsp90 function assists several stresses, including ER stress overload, the ROS over-generation, and a functional disorder of the intracellular proteins, ultimately leading to ER stress-induced apoptosis, if unfolding is overwhelming^54^,^55^,^56^,^57^. The unfolding protein response (UPR) is distinguished by three ER transmembrane receptors: protein kinase RNA-like ER kinase (PERK), activating transcription factor 6 (ATF6) and inositol-requiring enzyme 1 (IRE1), which involve both transcriptional and translational regulation of genes to expand the processing capacity of the ER and return this organelle to homeostasis^58^,^59^. If ER homeostasis is not restored, UPR promotes cell death. IRE1 is a transmembrane protein and cleaves microRNAs to control the levels of caspase family cell death proteases^60^. PERK is the major protein of the UPR and participates in ER stress-induced cell death, in part through the up-regulation of the proapoptotic CCAAT /enhancer binding protein homologous protein (Chop) expression and XIAP degradation^61^. In response to ER stress, ATF6, a transcriptional factor, is translocated to the Golgi compartment and cleaved by the action of two serine proteases, S1P and S2P. The cleaved ATF6 binds to ATF/cAMP response elements (CRE) and ER stress-response elements (ERSE-1) to activate the target genes, including Bip, GRP94 and Chop^58^. However, several lines of evidence suggested that Bip (Grp78) was not pivotal to switch the UPR on and off^58^,^62^. Moreover, Ca^2+^ trafficking in and out of ER regulates a diversity of cell responses and signaling transduction related with stress response, modulation of transcriptional processes and development^58^. In fact, it is known that Ca^2+^ release from the ER to accumulate in mitochondria could trigger a variety of signaling mechanisms to induce cell death mainly by Ca^2+^ -mediated mitochondrial cell death^63^. We observed that **1** treatment first stimulated reactive oxygen species (ROS) generation, perturbed the Bip/IRE1/PERK signal pathway and activated Grp94/ATF6/Chop signal pathway implicated in ER stress (Fig. 7B). In addition, large amount of Ca^2+^ release was observed which led to mitochondrial dysfunction-dependent apoptosis (Figs 2A,B and 7C).

Cumulative evidence suggests that several C-terminal Hsp90 inhibitors, as an alternatively clinical target, possess antiproliferative and apoptotic activities without eliciting heat shock response (HSR)^47^,^48^,^49^. Previous studies have indicated that activation of HSF 1 (Heat Shock Factor 1) is critical for the induction of HSR and may confer acquired resistance of Hsp90 inhibitors^64^,^65^. The master regulator, HSF 1, is involved with tumorigenesis and could possess potential target in cancer therapy^66^,^67^. Interestingly, compound **1**, a specific N-terminal Hsp90 inhibitor, activated Hsp70 induction and did not elicit the HSF 1 (Fig. 8C)^68^,^69^. It is worth noting that a cytosolic relay of Hsp70 and 90β monitors the folding trajectory of the serotonin transporter, as the principle target of antidepressant drugs^70^. This is presumably more important that the induction of Hsp70 by **1** compartmentalized in the cytosol of cells as demonstrated by immunofluorescent analysis (Fig. 8D). Accordingly, our results showed the potential merits of the scalarane sesterterpenoids as potential candidates for future clinical trials.

Nevertheless, some limitations to this present investigation were observed. First, the extremely small isolated quantity of **1** hindered the examination of its anti-leukemia effect in xenograft animal model. It also prevented the evaluation of **1** inhibitory activity on Hsp90 with cell-free system. Second, the transcription factors (HSF 1 and HIF 1) were blocked by **1** treatment; the factor of heat shock response that may contribute to the induction of Hsp70 protein has not been explored. Therefore, the key functions of Hsp70 induction should be further explored to identify the regulatory role in apoptosis process induced by this marine sesterterpenoid derivative.

In conclusion, we found that the treatment of cancer cells with the isolated scalarane sesterterpenoids (compounds **1** and **2**) and a meroditerpenoid (compound **3**) induced mitochondrial dysfunction, oxidative and ER stresses leading to apoptosis. Cell-free system and computational modeling using structure-function analysis further supported that these compounds can act as potential dual topoisomerase catalytic and Hsp90 inhibitors. The results confirmed a decrease in the expression of various Hsp90 client proteins in Molt 4 cells after treatment with **1**, the Hsp90 N-terminal inhibitor. Our studies provide further insights on the pro-apoptotic mechanism of these new sesterterpenoid derivatives, and their clinical potential as novel Hsp 90 and Topoisomerase II catalytic inhibitors to treat leukemia patients. Consequently, sesterterpenoids represent interesting molecular architectures which bind preferentially to proteins involved in tumorigenesis. Recent advances in sesterterpenoids synthesis^71^ open new avenues in their utilization in current therapeutic regimes to increase the effectiveness and decrease doses of these regimens leading to fewer side effects.

## Materials and Methods

### General Experimental Procedures

Infrared (IR) spectra were obtained on a Fourier-transform IR spectrophotometer Varian Digilab FTS 1000 (Varian Inc., Palo Alto, CA, USA). ^1^H and ^13^C NMR spectra were recorded on a Varian Unity INOVA 500 FT-NMR at 500 MHz and 125 MHz, respectively (Varian Inc., Palo Alto, CA, USA). Optical rotations were determined by a digital polarimeter Jasco P-1010 (Jasco Inc., Tokyo, Japan). Electrospray ionization mass spectrometry (ESIMS) analyses were performed on an APEX II Instrument (Bruker Daltonics, Billerica, MA, USA). Single-crystal X-ray analyses were performed on a Bruker APEX DUO diffractometer (Bruker Daltonics, Billerica, MA, USA). Column chromatography was performed with 230–400 mesh silica gel (Merck, AG, Darmstadt, Germany). TLC analyses were conducted on 0.2-mm-thick pre-coated Kieselgel 60 F254 plates (Merck, AG, Darmstadt, Germany), and the visualization of TLC spots was carried out by spraying the plate with 10% aqueous H_2_SO_4_ solution followed by heating. High-performance liquid chromatography (HPLC) was performed using a system consisting of a Hitachi L-7100 pump (Hitachi Ltd. Tokyo, Japan) and a Rheodyne 7725 injection port (Rheodyne LLC., Rohnert Park, CA, USA). A preparative normal phase column (φ 21.2 mm × 25 cm, silica gel 60, 5 μm) and a Supelco C_18_ column (φ 21.2 mm × 25 cm, 5 μm) (Supelco, Bellefonte, PA, USA) were used for HPLC. All methods were carried out in accordance with relevant guidelines and regulations.

### Animal Material

The specimen of *Carteriospongia* sp. was collected by scuba diving at a depth of 14 m from coral reefs off the coast of Tai-tung, Taiwan in March, 2013. Voucher specimen was deposited in the National Museum of Marine Biology and Aquarium, Taiwan (specimen No. 2013-03-SP-3). Taxonomic identification was performed by Li-Lian Liu of the National Sun Yat-sen University, Kaohsiung, Taiwan.

### Extraction and Isolation

*Carteriospongia* sp. (440 g fresh weight) was collected and freeze-dried. The freeze-dried material was minced and extracted exhaustively with EtOAc (6 × 2 L). The EtOAc extract was evaporated under reduced pressure to afford a residue (5 g), and the residue was subjected to column chromatography on silica gel, using *n*-hexane, *n*-hexane and EtOAc mixture of increasing polarity, and finally pure acetone to yield 8 fractions: Fr-1 (eluted by *n*-hexane:EtOAc, 50:1), Fr-2 (eluted by *n*-hexane:EtOAc, 25:1), Fr-3 (eluted by *n*-hexane:EtOAc, 10:1), Fr-4 (eluted by *n*-hexane:EtOAc, 5:1), Fr-5 (eluted by *n*-hexane:EtOAc, 2:1), Fr-6 (eluted by *n*-hexane:EtOAc, 1:1), Fr-7 (eluted by EtOAc) and Fr-8 (eluted by acetone). Fraction 2 (560.0 mg) was separated by normal-phase HPLC with gradient elution (*n*-hexane:EtOAc = 50:1 to 25:1) to yield 15 subfractions (2A-2O). Subfraction 2I was separated by normal phase HPLC (*n*-hexane:EtOAc = 40:1) to afford **3** (70.0 mg). Fraction 5 (320.0 mg) was further purified with silica gel (*n*-hexane:EtOAc = 4:1 to 1:1) to afford ten subfractions (5A–5J). Subfraction 5E was then separated by normal-phase HPLC (*n*-hexane:EtOAc = 3:1) to obtain **2** (6.0 mg). Subfraction 5G was separated by normal-phase HPLC (*n*-hexane:EtOAc = 3:1) to afford **1** (4.1 mg).

12*β*-(3′*β*-hydroxybutanoyloxy)-20,24-dimethyl-24-oxo-scalara-16-en-25-al (**1**): colorless oil; [*α*]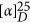 + 10.7 (*c* 0.41, CHCl_3_); IR (neat) *ν*_*max*_ 3448, 2963, 2931, 2875, 1718, 1666, 1374 and 1272 cm^−1^; ^13^C and ^1^H NMR data, see Table 1; ESIMS *m/z* 523[M + Na]^+^; HRESIMS *m/z* 523.3397 [M + Na]^+^ (calcd for C_31_H_48_O_5_Na, 523.3399).

12*β*-(3′*β*-hydroxypentanoyloxy)−20,24-dimethyl-24-oxo-scalara-16-en-25-al (**2**): colorless oil; 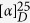 + 5.7 (*c* 0.50, CHCl_3_); IR (neat) *ν*_*max*_ 3448, 2930, 2854, 1730, 1666, 1388 and 1281 cm^−1^; ^13^C and ^1^H NMR data, see Table 1; ESIMS *m/z* 537[M + Na]^+^; HRESIMS *m/z* 537.3546 [M + Na]^+^(calcd for C_32_H_50_O_5_Na, 537.3550).

### Preparation of (*R*)- and (*S*)-MTPA Esters (2r and 2s)

Small amount of the compound **2** (2.5 mg) was divided, stored in two NMR tubes and dried under vacuum. Deuterated pyridine (0.60 mL) and (*R*)-MTPA-Cl (12 μL) were added to one of the NMR tubes. The reaction NMR tubes were permitted to stand at room temperature and monitored by 400 MHz NMR every hour. After 3 h, the reaction was found to be completed, and the ^1^H NMR data was obtained (400 MHz, in C_5_D_5_N). (*S*)-MTPA esters of **2** (**2s**) was obtained, and the ^1^H NMR data was analyzed. Similar to **2s**, (S)-MTPA-Cl (12 μL) and deuterated pyridine (0.60 mL) were reacted at room temperature for 3 h, to afford the (*R*)-MTPA ester derivatives (**2r**), in separate experiment, and the ^1^H NMR spectrum was measured with 400 MHz NMR in C_5_D_5_N. (*S*)-MTPA ester **2** (**2s**): ^1^H NMR (400 MHz, C_5_D_5_N): *δ* 9.928 (d, *J* = 3.6 Hz, 1H, H-25), 7.015 (s, 1H, H-16), 5.810 (t, *J* = 6.4 Hz, 1H, H-3′), 5.027 (dd, *J* = 10.8, 4.8 Hz, 1H, H-12), 3.253 (s, 1H, H-18), 2.255 (s, 3H, H-26), 1.134 (s, 3H, H-23), 1.110 (t, *J* = 6.0 Hz, 3H, H-5′), 0.882 (s, 3H, H-21), 0.793 (s, 3H, H-22), 0.776 (s, 3H, H-19), 0.743 (t, *J* = 7.2 Hz, 3H, H-25). (*R*)-MTPA ester **2** (**2r**): ^1^H NMR (400 MHz, C_5_D_5_N): *δ* 10.018 (d, *J* = 4.0 Hz, 1H, H-25), 7.065 (s, 1H, H-16), 5.833 (t, *J* = 6.4 Hz, 1H, H-3′), 5.123 (dd, *J* = 11.2, 4.0 Hz, 1H, H-12), 3.393 (s, 1H, H-18), 2.259 (s, 3H, H-26), 1.142 (s, 3H, H-23), 1.060 (t, *J* = 6.4 Hz, 3H, H-5′), 0.885 (s, 3H, H-21), 0.797 (s, 3H, H-22), 0.780 (s, 3H, H-19), 0.745 (t, *J* = 7.2 Hz, 3H, H-25).

### Bioassay Materials

The cell lines were obtained from the American Type Culture Collection (ATCC, Manassas, VA, USA). Cells were maintained in RPMI 1640 medium supplemented with 10% fetal calf serum, 2 mM glutamine and antibiotics (100 units/mL of penicillin and 100 μg/mL of streptomycin) at 37 °C in a humidified atmosphere of 5% CO_2_. RPMI 1640 medium, fetal calf serum (FCS), trypan blue, penicillin G and streptomycin were obtained from GibcoBRL (Gaithersburg, MD, USA). Dimethyl sulfoxide (DMSO), 3-(4,5-dimethylthiazol-2-yl)-2,5-diphenyl-tetrazolium bromide (MTT) and all other chemicals were purchased from Sigma-Aldrich (St. Louis, MO, USA). Antibodies against c-PARP, caspase 8, 9, 7, and 3, γH2AX, Bip, Grp 94, p-GSK 3β (Ser^9^), p-c-Raf, p70^S6K^, Hsp 90 and 70, Rb 2, MDM2, HIF 1, PERK and IRE 1 were purchased from Cell Signaling Technologies (Beverly, MA, USA). Antibodies against XIAP, NFκB (p65), GADD, CDK 4, HSF 1, ATF 6, and β-tubulin were obtained from Santa Cruz Biotechnology (Santa Cruz, CA, USA). Flou 3, JC-1 cationic dye and the carboxy derivative of fluorescein (carboxy-H_2_DCFDA) were purchased from Molecular Probes and Invitrogen technologies (Carlsbad, CA, USA). Anti-mouse and rabbit IgG peroxidase-conjugated secondary antibody were purchased from Pierce (Rockford, IL, USA). The Annexin V-FITC/PI (propidium iodide) kit was from Strong Biotech Corporation (Taipei, Taiwan). The Hybond ECL transfer membrane and ECL Western blotting detection kits were obtained from Amersham Life Sciences (Amersham, UK).

### Annexin V/PI Apoptosis Assay

The externalization of phosphatidylserine (PS) and membrane integrity were quantified using an annexin V- FITC staining kit^72^. In brief, 10^6^ cells were grown in 35 mm diameter plates and were labeled with annexin V-FITC (10 μg/mL) and PI (20 μg/mL) prior to harvesting. After labeling, all plates were washed with a binding buffer and harvested. Cells were resuspended in the binding buffer at a concentration of 2 × 10^5^ cells/mL before analysis by flow cytometer FACS-Calibur (Becton-Dickinson, San Jose, CA, USA) and CellQuest software. Approximately 10,000 cells were counted for each determination.

### Determination of ROS Generation, Calcium accumulation, and MMP Disruption

These assays were performed as described previously^38^. MMP disruption, calcium accumulation and ROS generation were detected with JC-1 cationic dye (5 μg/mL), the fluorescent calcium indicator (Fluo 3, 5 mM) and the carboxy derivative of fluorescein (carboxy-H_2_DCFDA, 1.0 mM), respectively. In brief, the treated cells were labeled with a specific fluorescent dye for 30 min. After labeling, cells were washed with PBS and resuspended in PBS at a concentration of 1 × 10^6 ^cells/mL before analysis via flow cytometry.

### Assay of Topoisomerase II Catalytic Inhibitors and Poisons

The assay was performed as described previously^35^,^38^. Standard relaxation reaction mixtures (20 μL) containing 50 mM Tris–HCl (pH 8.0), 10 mM MgCl_2_, 200 mM potassium glutamate, 10 mM dithiothreitol, 50 μg/mL bovine serum albumin, 1 mM ATP, 0.3 μg of pHOT1 plasmid DNA, two units of human topoisomerase II (Topogen, Columbus, OH, USA), and the indicated concentrations of etoposide and the compounds were incubated at 37 °C for 30 min. Reactions were terminated by adding 2 μL of 10% SDS to facilitate trapping the enzyme in a cleavage complex, followed by the addition of 2.5 μL of proteinase K (50 μg/mL) to digest the bound protein (incubated at 37 °C for 15 min) and finally by adding 0.1 volume of the sample loading dye. The DNA products were analyzed via electrophoresis through vertical 2% agarose gels at 2 voltage/cm in 0.5 × TAE buffer. Gels were stained with ethidium bromide and photographed using an Eagle Eye II system (Stratagene, La Jolla, CA, USA). The quantitative analysis of the DNA Topo II activity was performed as described previously^73^. The gels were directly scanned with image analyzer, and the area representing supercoiled DNA calculated to evaluate the concentration that the compounds caused 50% inhibition (IC50) of Topo II activity.

### Neutral Comet Assay for Detection of DNA Double-strand Breaks (DSBs)

The assay was carried out using a CometAssayTM Kit (Trevigen, Gaithersburg, MD, USA) following the manufacturer’s protocol for the neutral Comet assay. Briefly, cancer cells (2 × 10^5 ^cells/mL) were treated with compound **1** (0.0625 μg/mL) at the indicated time. Cells were combined with 1% low melting point agarose at a ratio of 1:10 (v/v) and immediately 75 μL of the mixture was pipetted onto CometSlide^TM^ and allowed to set at 4 °C in the dark. The slides were immersed in ice-cold lysis solution (Trevigen) for 30 to 60 min. The slides were placed in a horizontal electrophoresis apparatus and electrophoresed in 1X TBE (90 mM Tris-HCl, 90 mM boric acid, and 2 mM EDTA, pH 8.0) at 20 V for 10 min. The samples were then fixed in 70% ethanol and dried before stained with 1:10,000 SYBR Green I (Trevigen) to visualize cellular DNA. The fluorescence images were analyzed using the TriTek Comet Image program to circumscribe the “head” and the “tail” regions of each comet and the integrated fluorescence values of each defined area were recorded. The comet length was measured from the trailing edge of the nucleus to the leading edge of the tail. This length was indicative of the extent of DNA damage. Calculations were averaged per replicate.

### Western Blotting Analysis

Cell lysates were prepared by treating the cells for 30 min in RIPA lysis buffer, 1% Nonidet P-40, 0.5% sodium deoxycholate, 0.1% sodium dodecyl sulphate (SDS), 1 mM sodium orthovanadate, 100 μg/mL phenylmethylsulfonyl fluoride and 30 μg/mL aprotinin) (all chemicals were from Sigma)^38^. The lysates were centrifuged at 20,000 × *g* for 30 min, and the protein concentration in the supernatant was determined using a BCA protein assay kit (Pierce). Equal amounts of proteins were respectively separated by 7.5%, 10% or 12% of SDS-polyacrylamide gel electrophoresis and then were electrotransferred to a PVDF membrane. The membrane was blocked with a solution containing 5% non-fat dry milk TBST buffer (20 mM Tris-HCl, pH 7.4, 150 mM NaCl and 0.1% Tween 20) for 1 h and washed with TBST buffer. The protein expressions were monitored by immunoblotting using specific antibodies. These proteins were detected by an enhanced chemiluminescence kit (Pierce).

### Immunofluorescence Analysis

After treatment with the tested compound, cells were fixed with 4% paraformaldehyde in 50 mM HEPES buffer (pH 7.3) for 30 min, and permeabilized for 20 min with 0.2% Trition X-100 in PBS (pH 7.4). To prevent non-specific protein binding, cells were incubated with 5% BSA in PBS containing 0.05% Trition X-100 (T-PBS) for 1 h at room temperature. Cells were then incubated with the primary Hsp70 antibodies (1: 500) for 2 h and further with secondary antibodies (Alexa Fluor 586-conjugated goat anti-mouse IgG (H + L) (Life Technologies, Carlsbad, CA, USA) diluted at 1:1000 for 1 h at room temperature. After washing with PBS, cells were observed under a FV1000 confocal laser scanning microscope (Olympus, Tokyo, Japan).

### Human Leukemia Molt 4 Cells Xenograft Animal Model

Establishment of nude mice with xenografts was performed as described previously^38^. Six-week-old male immunodeficient athymic mice were purchased from the National Laboratory Animal and Research Center (Taipei, Taiwan). All of the animals were maintained under standard laboratory conditions (temperature 24–26 °C, 12–12 h dark-light circle) and fed with laboratory diet and water. This study was approved by the Animal Care and Treatment Committee of Kaohsiung Medical University (IACUC Permit Number 101136). All experiments were conducted in strict accordance with the recommendations in the Guide for the Care and Use of Laboratory Animals of the National Institutes of Health, and all efforts were made to minimize animal stress/distress. Molt 4 cells (1 × 10^6^) resuspended in 0.2 mL PBS were injected *s.c*. into the right flank of each mouse, and tumor growth was monitored every day. Fourteen days after tumor cell injection, mice with confirmed tumor growth were randomly divided into two groups. Compound **3** (1.14 μg/g) was intraperitoneally administered to the treatment group, and the control group received solvent only. Compound **3** was administrated every other day for 33 days. Animals were sacrificed by carbon dioxide. Tumor size was measured three times a week using calipers and tumor volumes were calculated according to the standard formula: width^2^ × length/2.

### Molecular Modeling Assay

The molecular docking was performed by Autodock 4.2 with Lamarckian Genetic Algorithm^74^. The target macromolecule, Hsp90 protein (PDB ID: 1YET), was obtained from the Protein data bank (http://www.rcsb.org/pdb/home/home.do)^75^. The co-crystalized protein substrates, including ligands, water and small molecules were removed, and the Polar hydrogens and Kallman united atom charges were added to the protein for docking calculation by AutoDock Tool 1.5.4 interfaces (ADT)^76^. The ligands were optimized with MMFF94 force field by ChemBio3D software (version 11.0; Cambridge Soft Corp.). Polar hydrogens and Gasteiger charges were also added to the ligand for docking study by ADT. The Grid box calculated by AutoGrid program was centered at the activity site of Hsp90 with dimensions 56 × 56 × 56 Å grid points at spacing of 0.375Å and its size is big enough to allow the ligand move freely in the search space. All docking parameters were set to default except for the following parameter: maximum number of energy evaluation increase to 25,000,000 per run. The docking results were analyzed by ADT and shown by Accelrys Discovery Studio v3.5 client software (Accelrys Inc, San Diego, CA (2005)).

### Statistics

The results were expressed as mean ± standard deviation (SD). Comparison in each experiment was performed using an unpaired Student’s *t*-test and a *p* value of less than 0.05 was considered to be statistically significant.

## Additional Information

**How to cite this article**: Lai, K.-H. *et al*. Antileukemic Scalarane Sesterterpenoids and Meroditerpenoid from *Carteriospongia (Phyllospongia)* sp., Induce Apoptosis via Dual Inhibitory Effects on Topoisomerase II and Hsp90. *Sci. Rep*. **6**, 36170; doi: 10.1038/srep36170 (2016).

**Publisher’s note:** Springer Nature remains neutral with regard to jurisdictional claims in published maps and institutional affiliations.

## Supplementary Material

Supplementary Information

## Figures and Tables

**Table 1:** ^1^H and ^13^C NMR data of 1 and 2 (^1^H: 500 MHz in CDCl_3_; ^13^C: 125 MHz in CDCl_3_).

	1		2	
position	*δ*_C_ type	*δ*_H_ (*J* in Hz)	*δ*_C_	*δ*_H_ (*J* in Hz)
1	40.1, CH_2_	0.88, 1.64 m	40.1	0.87, 1.64 m
2	18.1, CH_2_	0.84 m	18.1	0.84, 1.44 m
3	36.5, CH_2_	0.87 m; 1.66 br s	36.5	0.87, 1.66 m
4	36.1, C		36.1	
5	58.4, CH	0.87 m	58.4	0.88 m
6	17.9, CH_2_	0.85 m	17.9	0.87 m
7	41.9, CH_2_	1.75 m	42.0	1.74 m
8	37.5, C		37.5	
9	58.0, CH	0.99 m	58.0	1.01 br s
10	37.4, C		37.4	
11	23.3, CH_2_	1.40 q (12.5);	23.2	1.88 m
		1.87 dd (12.5, 4.0)		
12	82.5, CH	4.86 dd (11.0, 4.5)	82.5	4.87 dd (11.0, 4.5)
13	41.7, C		41.7	
14	53.0, CH	1.21 m	53.0	1.22 m
15	23.6, CH_2_	2.34 br t	23.6	2.34 m
16	142.9, CH	7.06 s	142.8	7.06 s
17	138.4, C		138.5	
18	61.2, CH	3.15 s	61.2	3.17 s
19	28.4, CH_3_	0.80 s	28.4	0.79 s
20	24.4, CH_2_	1.15 q (7.5); 1.52 m	24.4	1.15, 1.50 m
21	16.9, CH_3_	0.96 s	16.9	0.96 s
22	17.3, CH_3_	0.85 s	17.3	0.85 s
23	10.9, CH_3_	0.98 s	10.9	0.98 s
24	198.2, C		198.2	
25	201.1, CH	9.64 d (3.5)	201.2	9.65 d (3.0)
26	25.0, CH_3_	2.28 s	25.0	2.28 s
27	8.6, CH_3_	0.74 t (7.5)	8.6	0.74 t (7.5)
1′	172.1, C		172.3	
2′	43.4, CH_2_	2.42 m	41.6	1.14, 1.36 m
3′	64.3, CH	4.22 t (9.0)	69.4	3.96 t (11.0)
4′	22.5, CH_3_	1.24 d (6.5)	29.5	2.32 m
5′			9.9	0.96 t (4.0)
