# Supplementary material for: Antileukemic Scalarane Sesterterpenoids and Meroditerpenoid from Carteriospongia (Phyllospongia) sp., Induce Apoptosis via Dual Inhibitory Effects on Topoisomerase II and Hsp90
Source: Sci Rep. 2016 Oct 31;6:36170. doi: 10.1038/srep36170 (PMC5086919; doi:10.1038/srep36170)
Supplement: Supplementary Information [file srep36170-s1.pdf]

Supplementary Information

Antileukemic Scalarane Sesterterpenoids and Meroditerpenoid from *Carteriospongia (Phyllospongia)* sp., Induce Apoptosis via Dual Inhibitory Effects on Topoisomerase II and Hsp90

Kuei-Hung Lai[1,2,3,4,+], Yi-Chang Liu[5,6,+], Jui-Hsin Su[1,2], Mohamed El-Shazly[7], Chih-Fung Wu[8], Ying-Chi Du[1,2,3], Yu-Ming Hsu[3], Juan-Cheng Yang[9,10], Ming-Kai Weng[1], Chia-Hua Chou[1,2], Guan-Yu Chen[9,10], Yu-Cheng Chen[11] & Mei-Chin Lu[1,2,*]

[1]Graduate Institute of Marine Biology, National Dong Hwa University, Pingtung, 944, Taiwan. [2]National Museum of Marine Biology & Aquarium, Pingtung 944, Taiwan. [3]Graduate Institute of Natural Products, College of Pharmacy, Kaohsiung Medical University, Kaohsiung 807, Taiwan. [4]Division of Pharmacognosy, Department of Medicinal Chemistry, Uppsala University, Uppsala, Sweden. [5]Division of Hematology-Oncology, Department of Internal Medicine, Kaohsiung Medical University Hospital, Kaohsiung, 807, Taiwan. [6]Department of Internal Medicine, Faculty of Medicine, College of Medicine, Kaohsiung Medical University, Kaohsiung, 807, Taiwan. [7]Department of Pharmacognosy and Natural Products Chemistry, Faculty of Pharmacy, Ain-Shams University, Organization of African Unity Street, Abassia, Cairo 11566, Egypt. [8]Division of Surgical Oncology, Department of Surgery, Kaohsiung Medical University Hospital, Kaohsiung 807, Taiwan. [9]School of Pharmacy, College of Pharmacy, China Medical University, Taichung, Taiwan. [10]Chinese Medicine Research and Development Center, China Medical University Hospital, Taichung, Taiwan. [11]The Ph.D. Program for Cancer Biology and Drug Discovery, China Medical University and Academia Sinica, Taichung, Taiwan. [+]K.-H. L. and Y.-C. L. contributed equally to this study. [*]Correspondence and requests for materials should be addressed to M.-C. Lu (jinx6609@yahoo.com.tw).

Table of Contents

Figure S1: ^{1}H NMR (500 MHz, $CDCl_3$) spectrum of **1**. --- 3

Figure S2: ^{13}C NMR (125 MHz, $CDCl_3$) spectrum of **1**. -- 4

Figure S3: DEPT NMR (125 MHz, $CDCl_3$) spectrum of **1**. -- 5

Figure S4: COSY NMR (500 MHz, $CDCl_3$) spectrum of **1**. --- 6

Figure S5: NOESY NMR (500 MHz, $CDCl_3$) spectrum of **1**. --- 7

Figure S6: HSQC NMR (500 MHz, $CDCl_3$) spectrum of **1**. --- 8

Figure S7: HMBC NMR (500 MHz, $CDCl_3$) spectrum of **1**. --- 9

Figure S8: HRESIMS spectrum of **1**. --- 10

Figure S9: IR spectrum of **1**. --- 11

Figure S10: ^{1}H NMR (500 MHz, $CDCl_3$) spectrum of **2**. --- 12

Figure S11: ^{13}C NMR (125 MHz, $CDCl_3$) spectrum of **2**. -- 13

Figure S12: DEPT NMR (125 MHz, $CDCl_3$) spectrum of **2**. --- 14

Figure S13: COSY NMR (500 MHz, $CDCl_3$) spectrum of **2**. --- 15

Figure S14: NOESY NMR (500 MHz, $CDCl_3$) spectrum of **2**. --- 16

Figure S15: HSQC NMR (500 MHz, $CDCl_3$) spectrum of **2**. --- 17

Figure S16: HMBC NMR (500 MHz, $CDCl_3$) spectrum of **2**. --- 18

Figure S17: HRESIMS spectrum of **2**. -- 19

Figure S18: IR spectrum of **2**. --- 20

Figure S19: Values of $\Delta\delta_{S\text{-}R}$ of the MTPA esters of **2**. --- 21

Figure S20: The full length gel of Topo IIα activity. --- 22

Figure S21: The full length blots of caspase 9, 8 and 3 expressions. ----------------------------------- 23

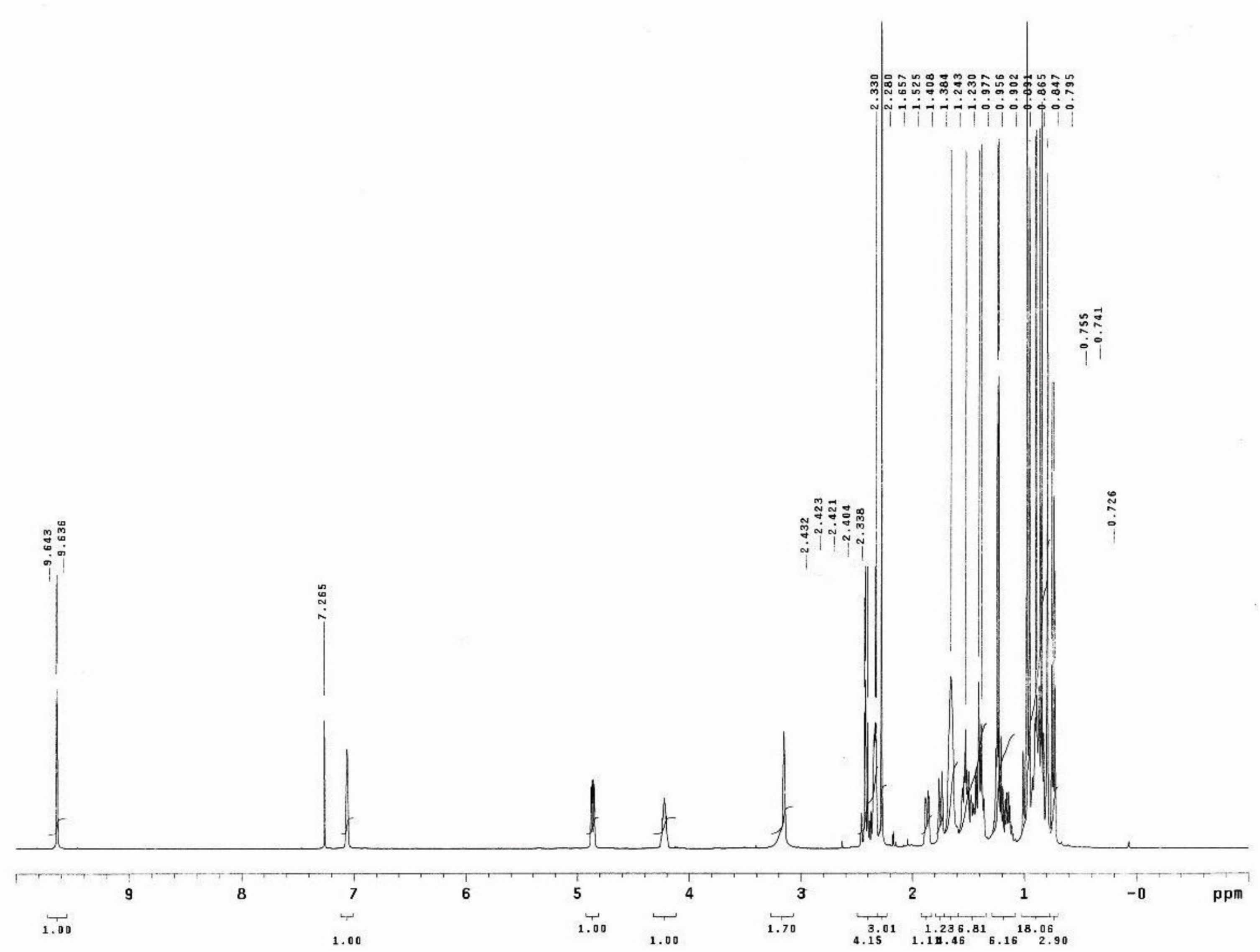

Figure S1: ^{1}H NMR (500 MHz, $CDCl_3$) spectrum of **1**.

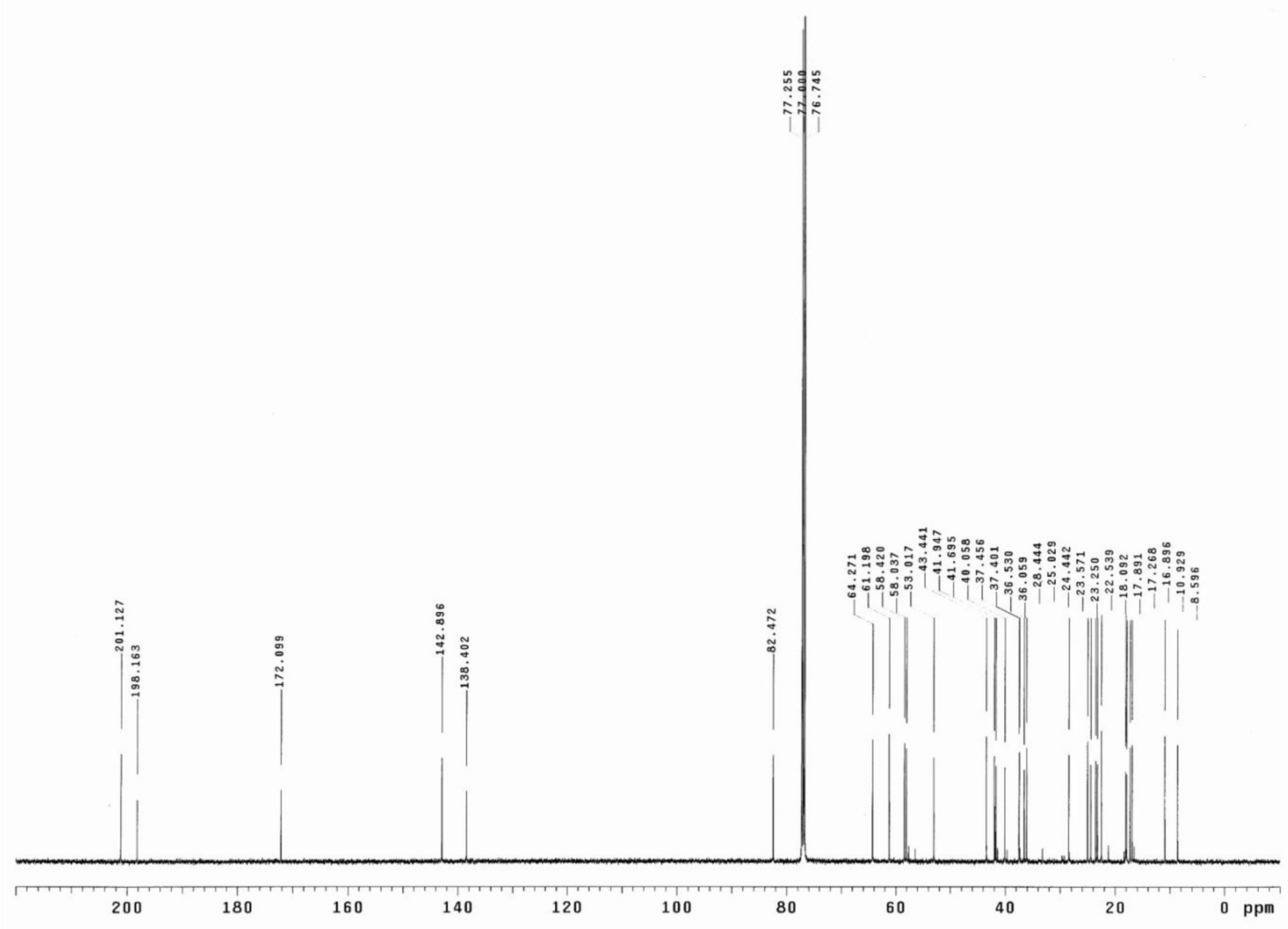

Figure S2: ^{13}C NMR (125 MHz, $CDCl_3$) spectrum of **1**.

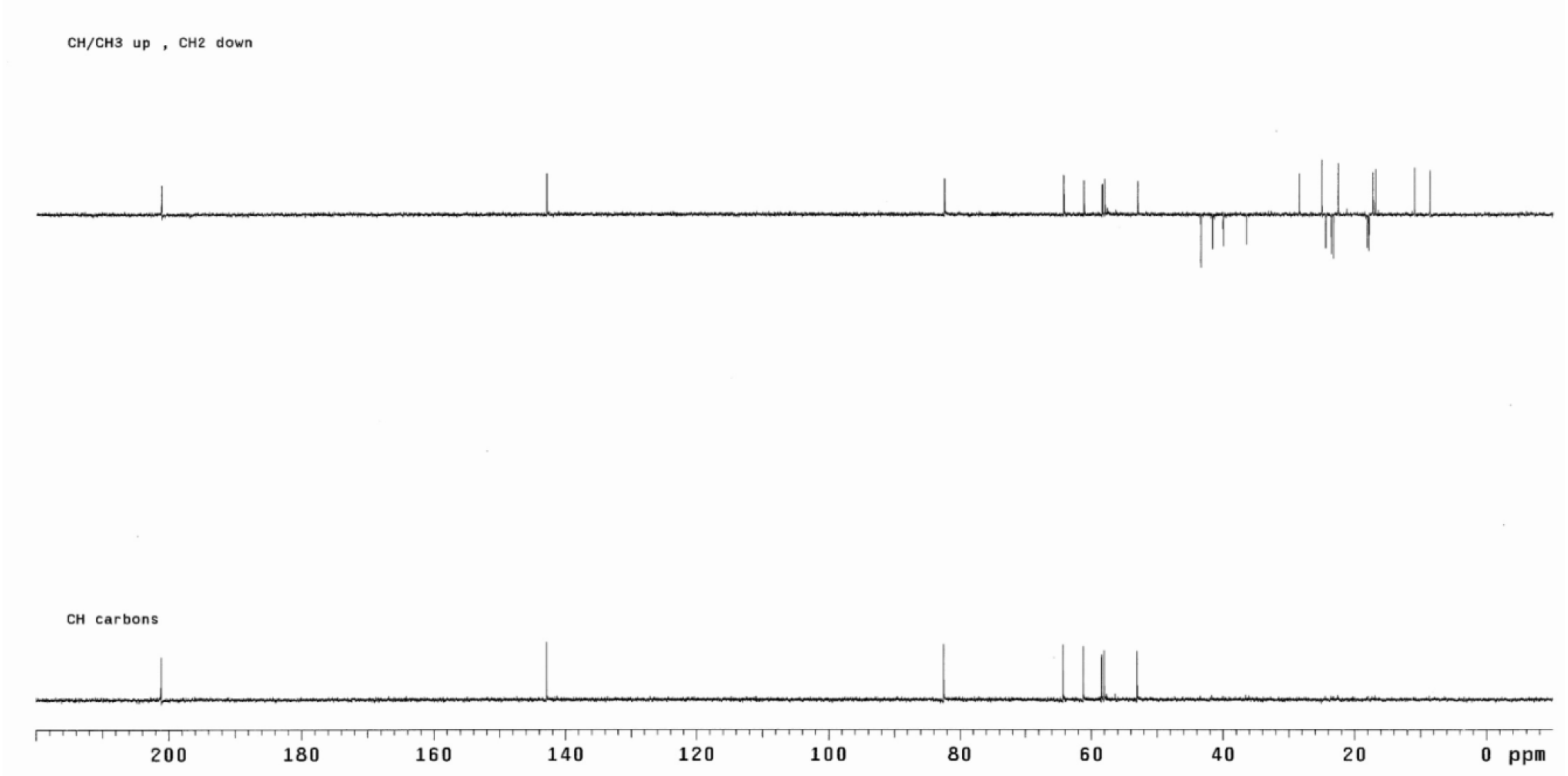

Figure S3: DEPT NMR (125 MHz, $CDCl_3$) spectrum of **1**.

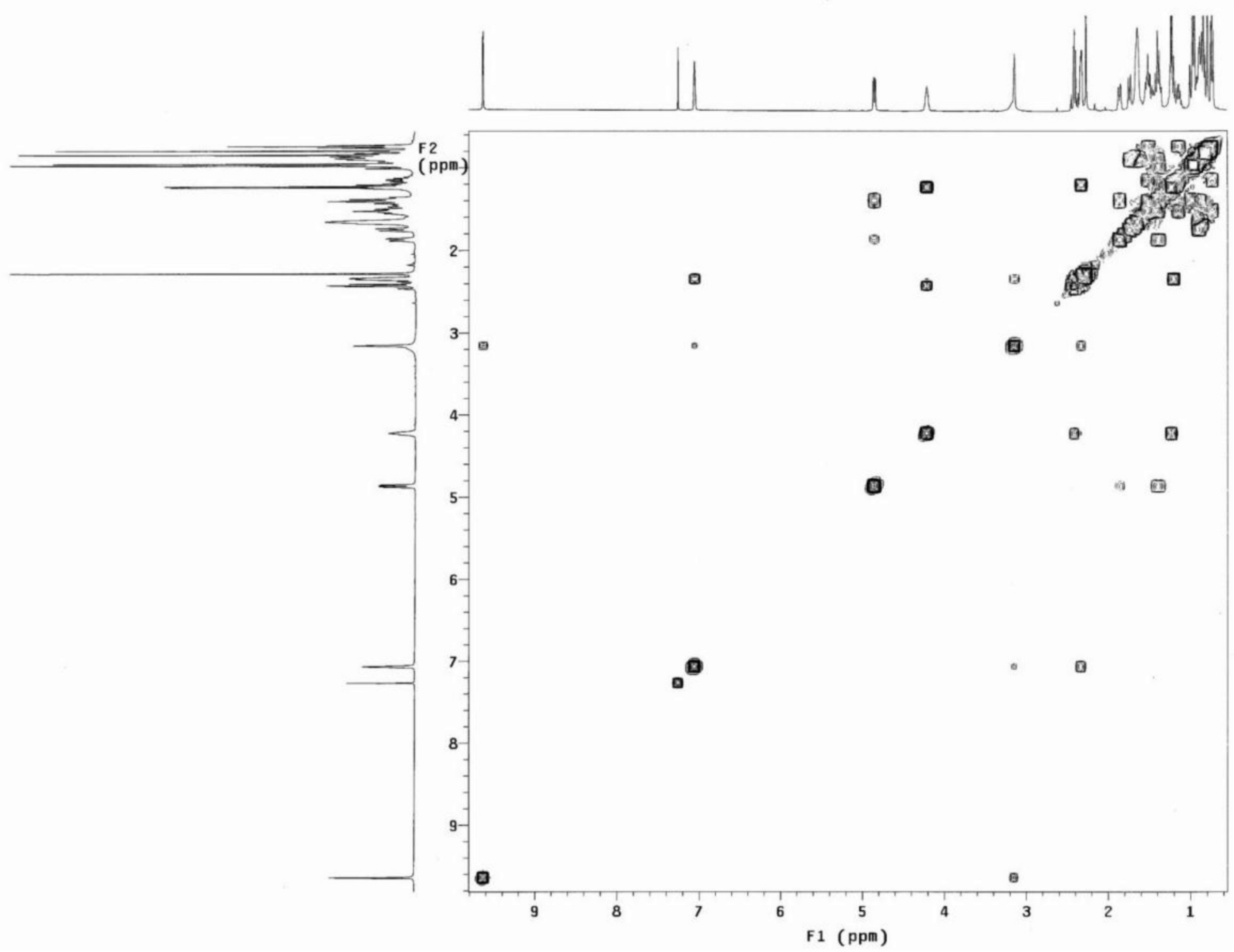

Figure S4: COSY NMR (500 MHz, $CDCl_3$) spectrum of **1**.

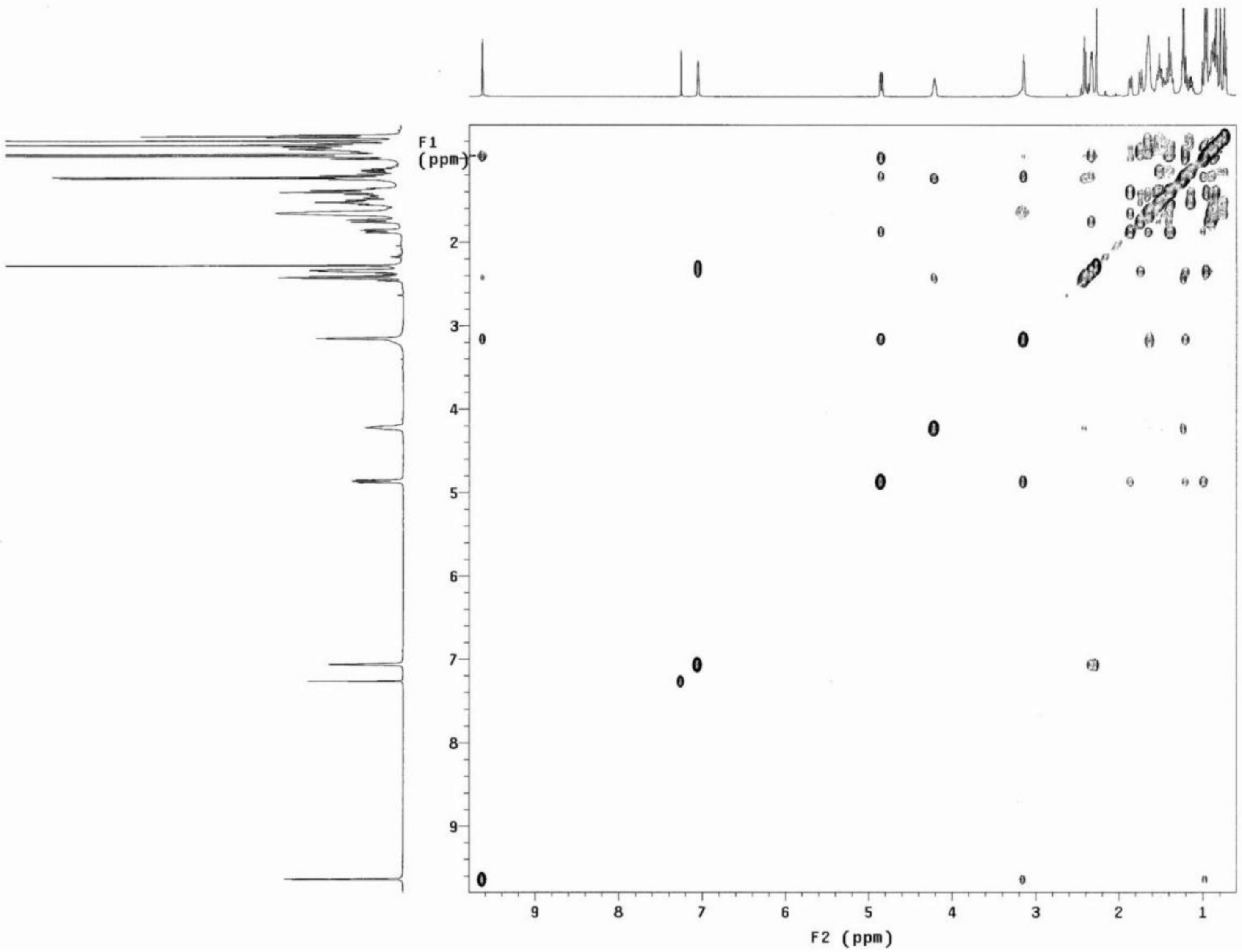

Figure S5: NOESY NMR (500 MHz, $CDCl_3$) spectrum of **1**.

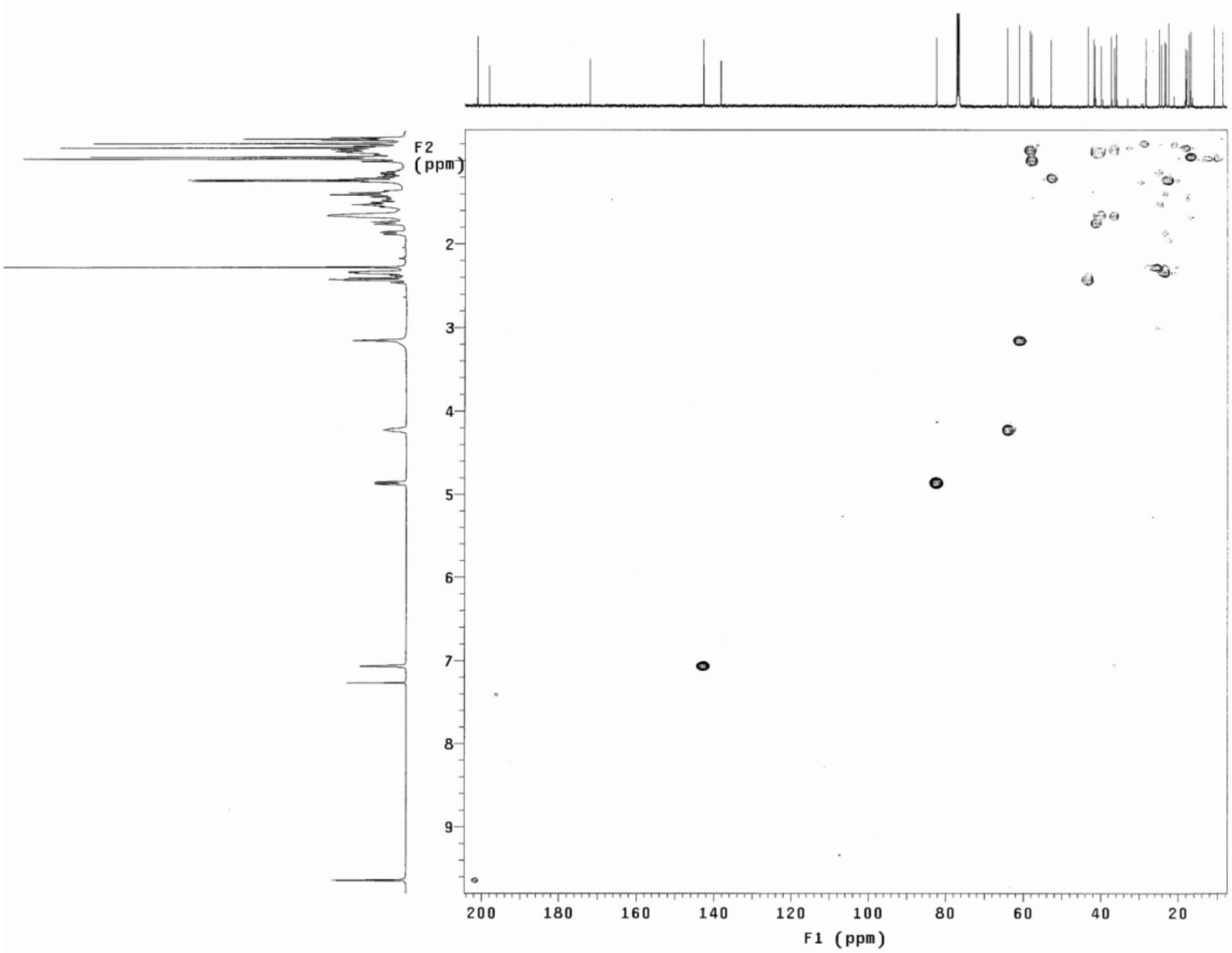

Figure S6: HSQC NMR (500 MHz, $CDCl_3$) spectrum of **1**.

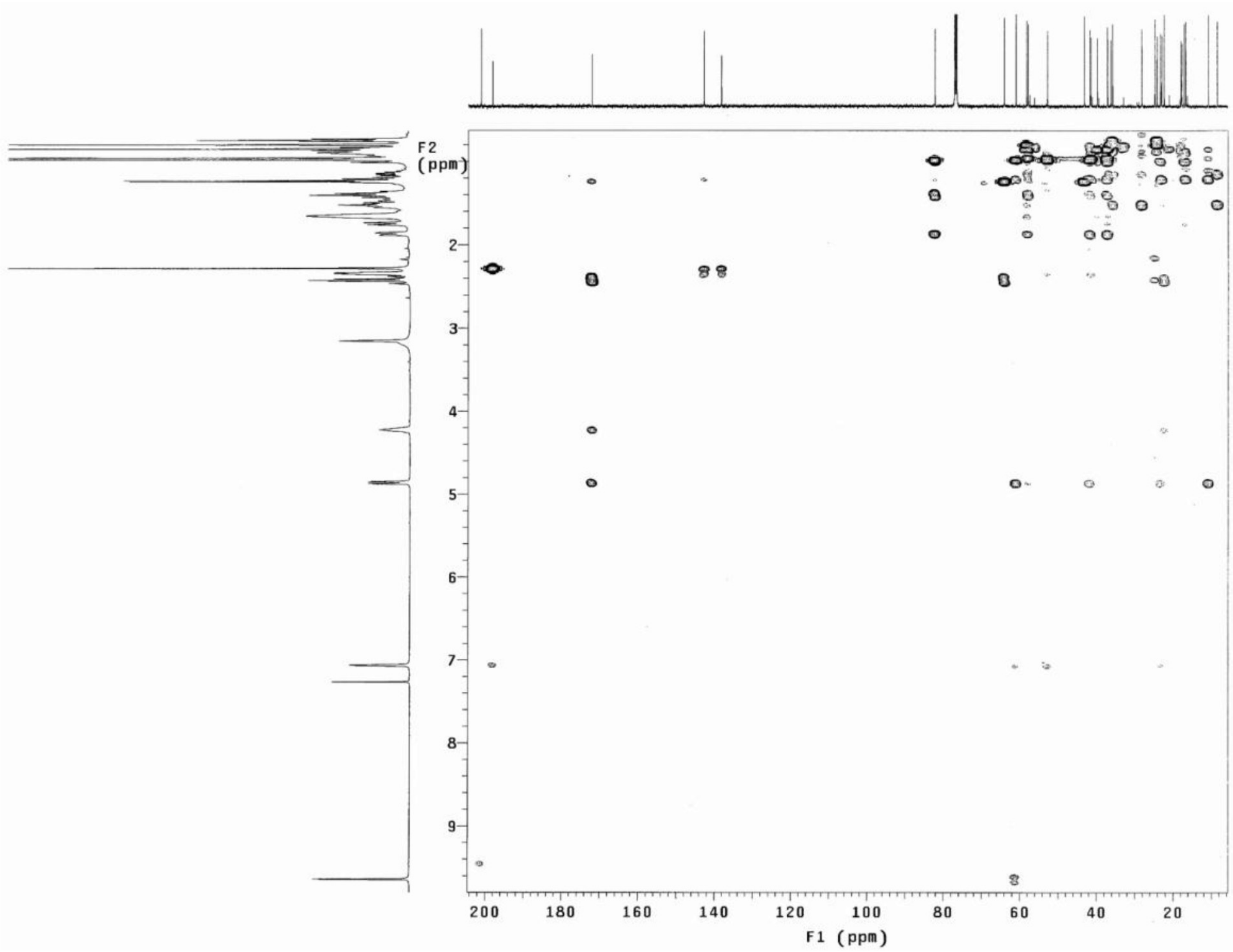

Figure S7: HMBC NMR (500 MHz, $CDCl_3$) spectrum of **1**.

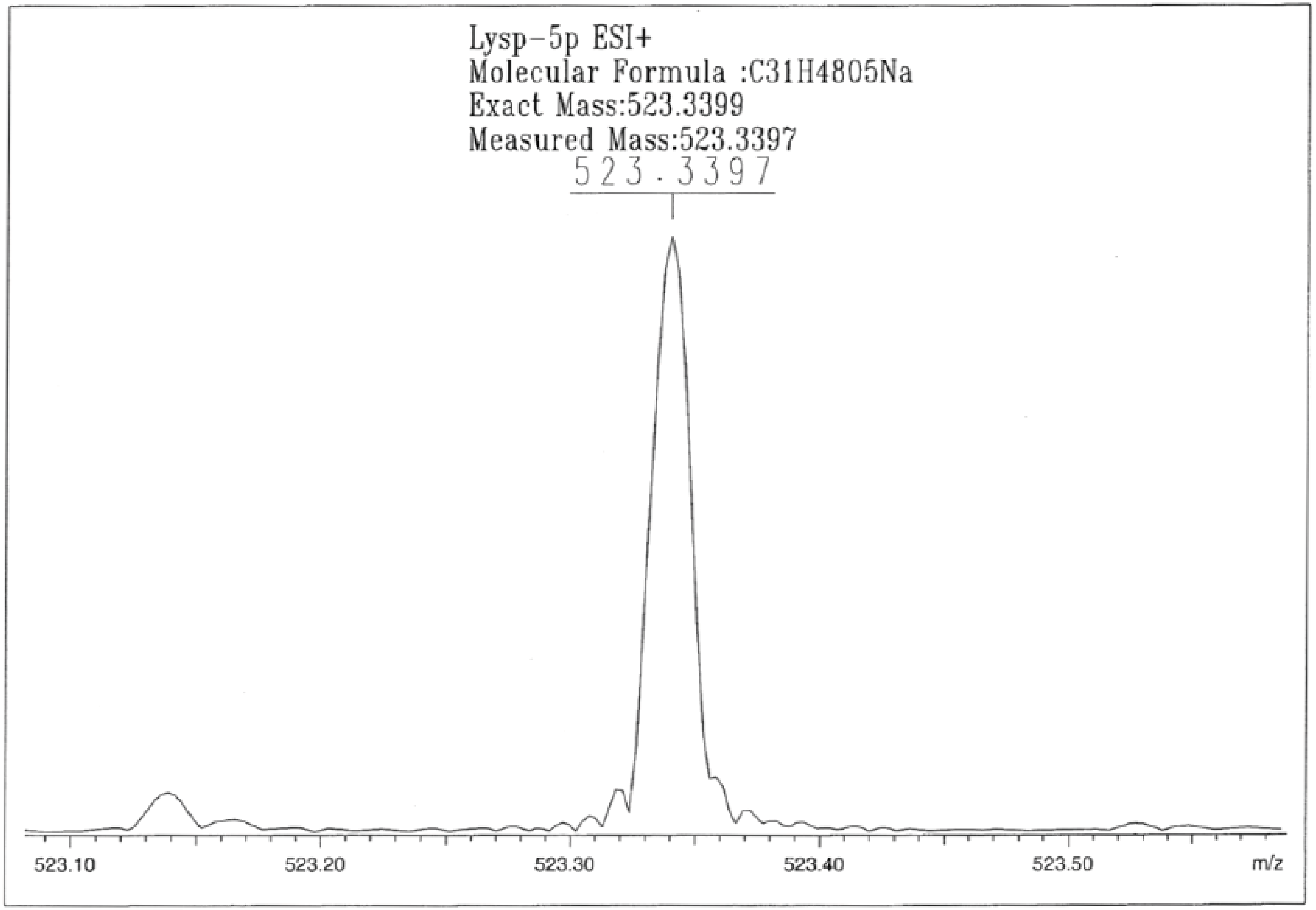

Figure S8: HRESIMS spectrum of **1**.

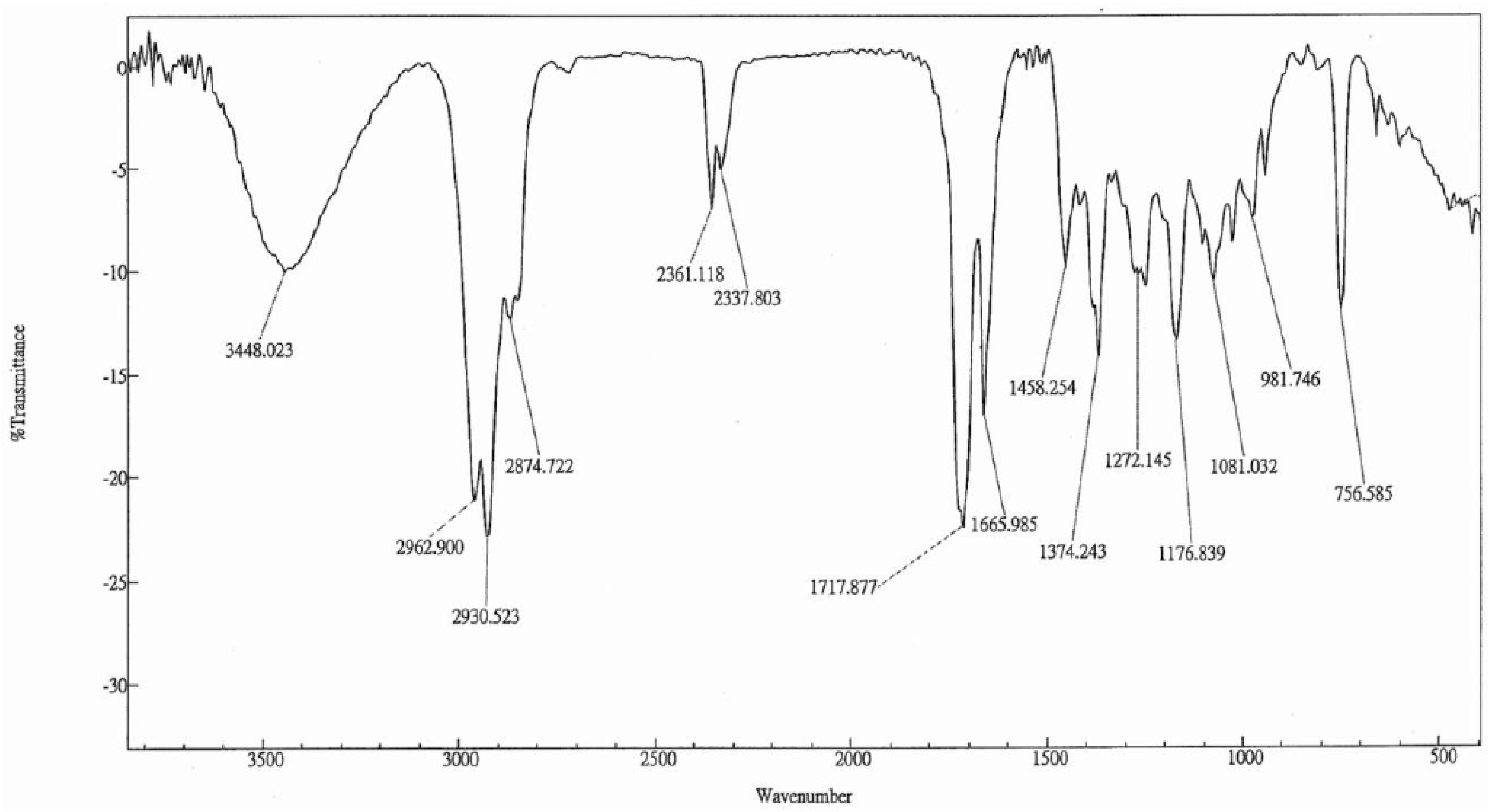

Figure S9: IR spectrum of **1**.

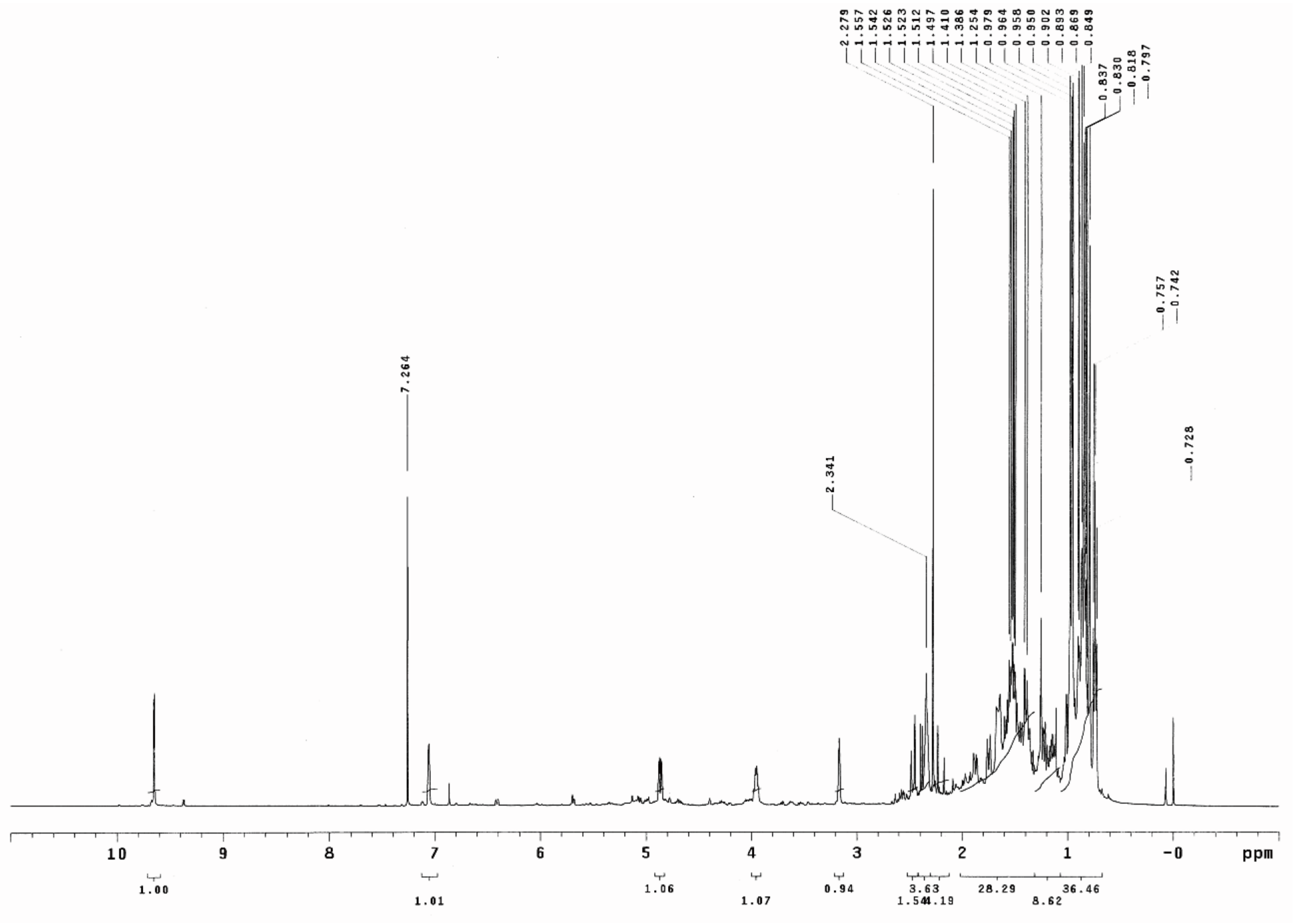

Figure S10: ^{1}H NMR (500 MHz, $CDCl_3$) spectrum of **2**.

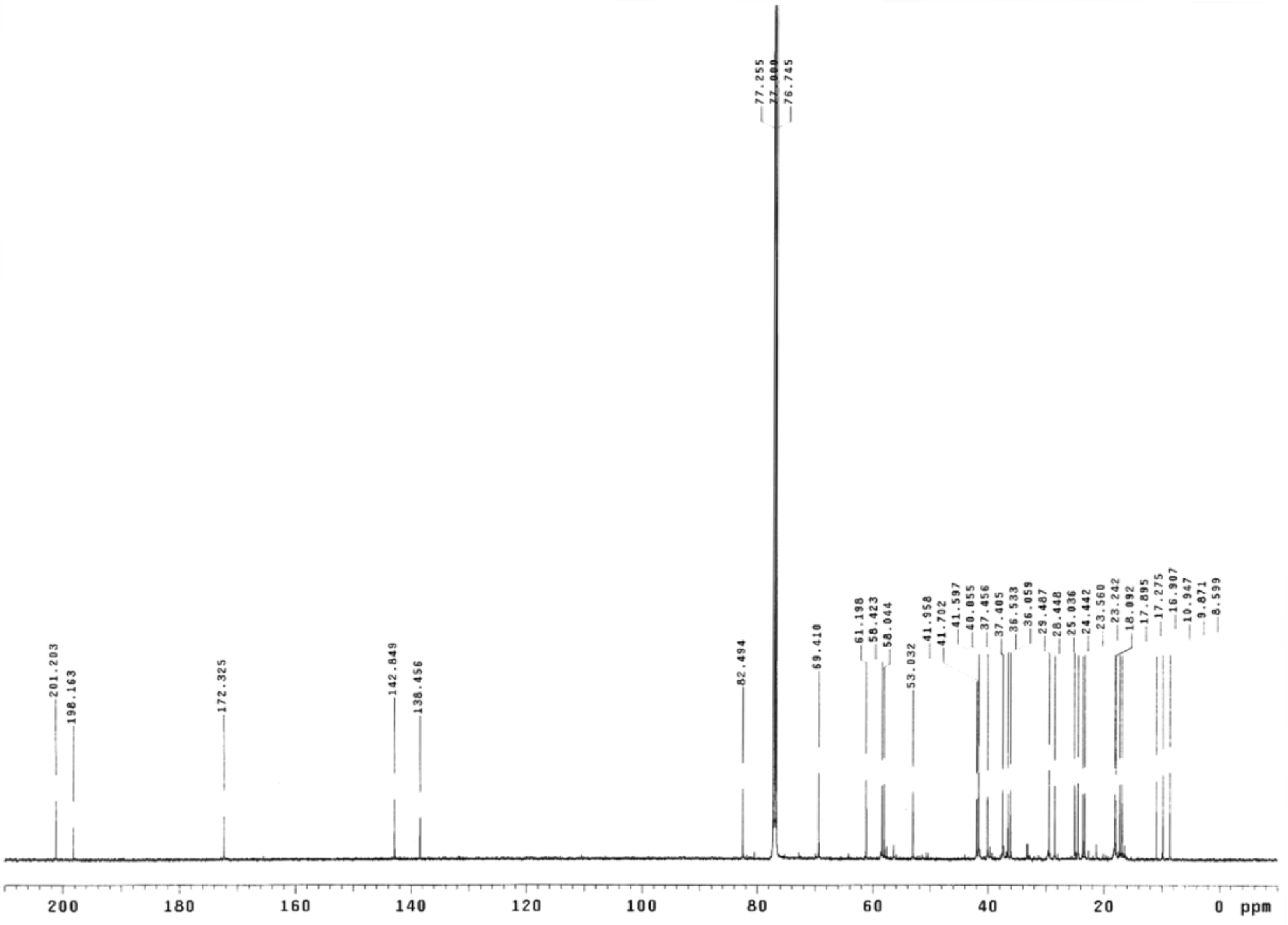

Figure S11: ^{13}C NMR (125 MHz, $CDCl_3$) spectrum of **2**.

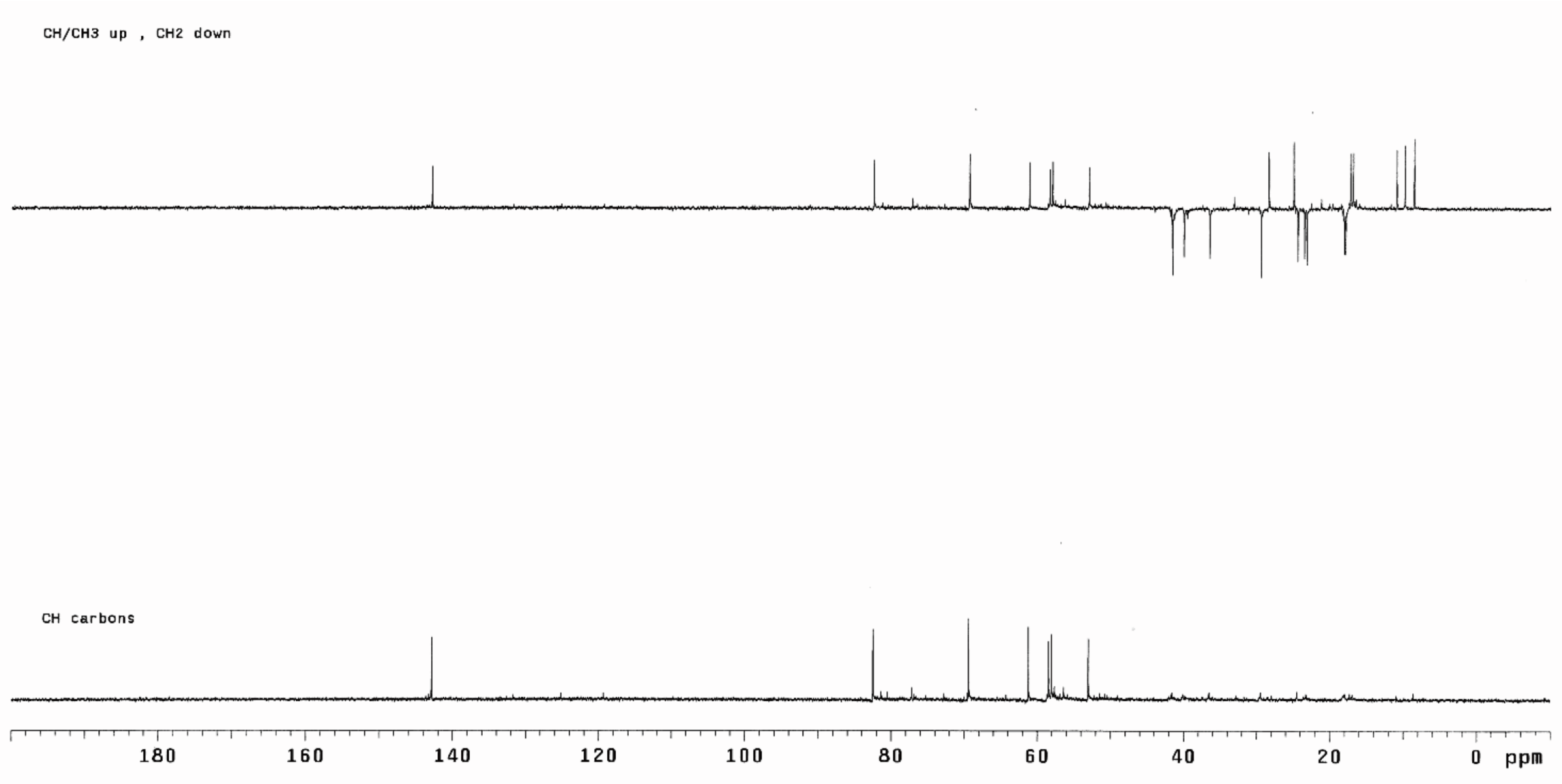

Figure S12: DEPT NMR (125 MHz, $CDCl_3$) spectrum of **2**.

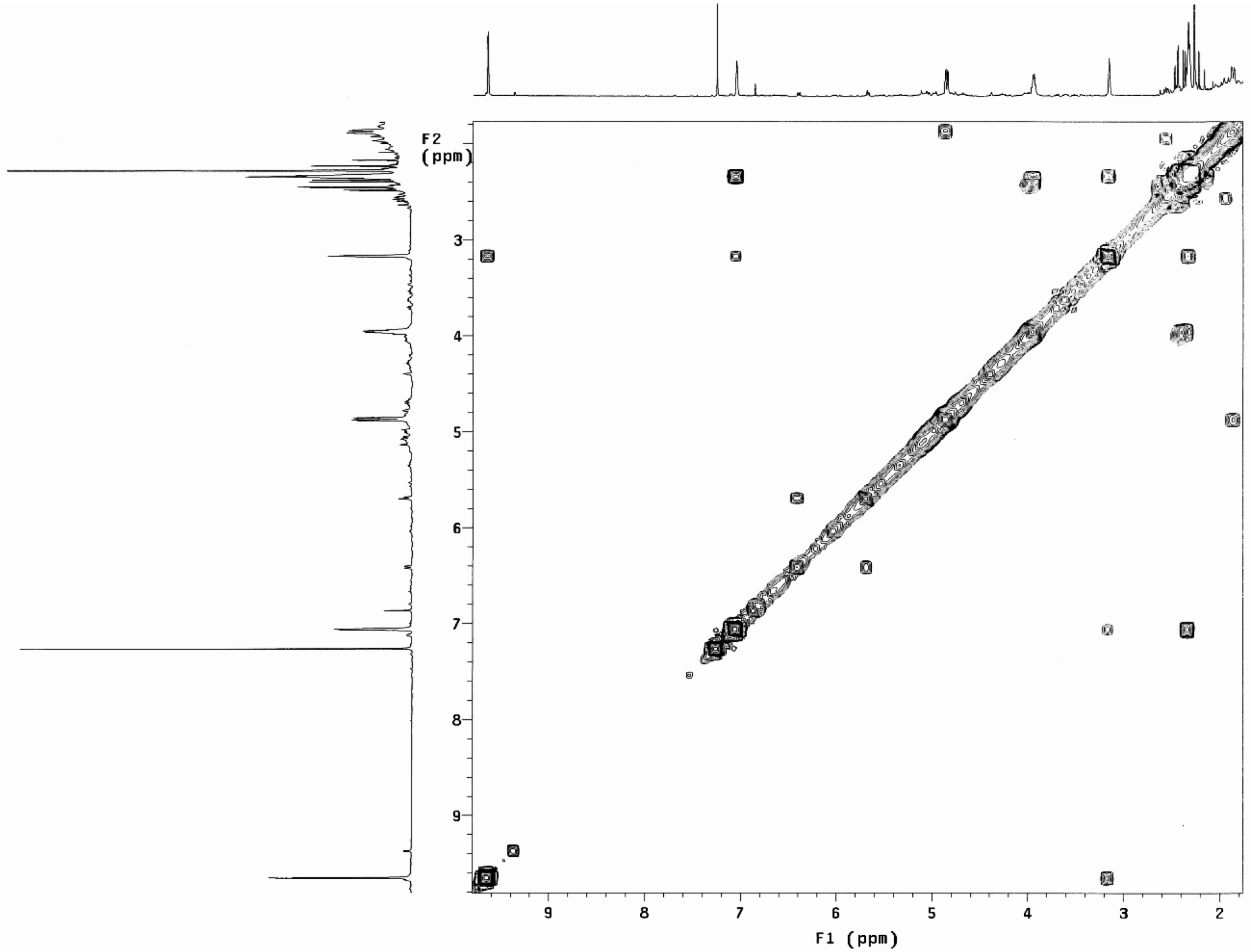

Figure S13: COSY NMR (500 MHz, $CDCl_3$) spectrum of **2**.

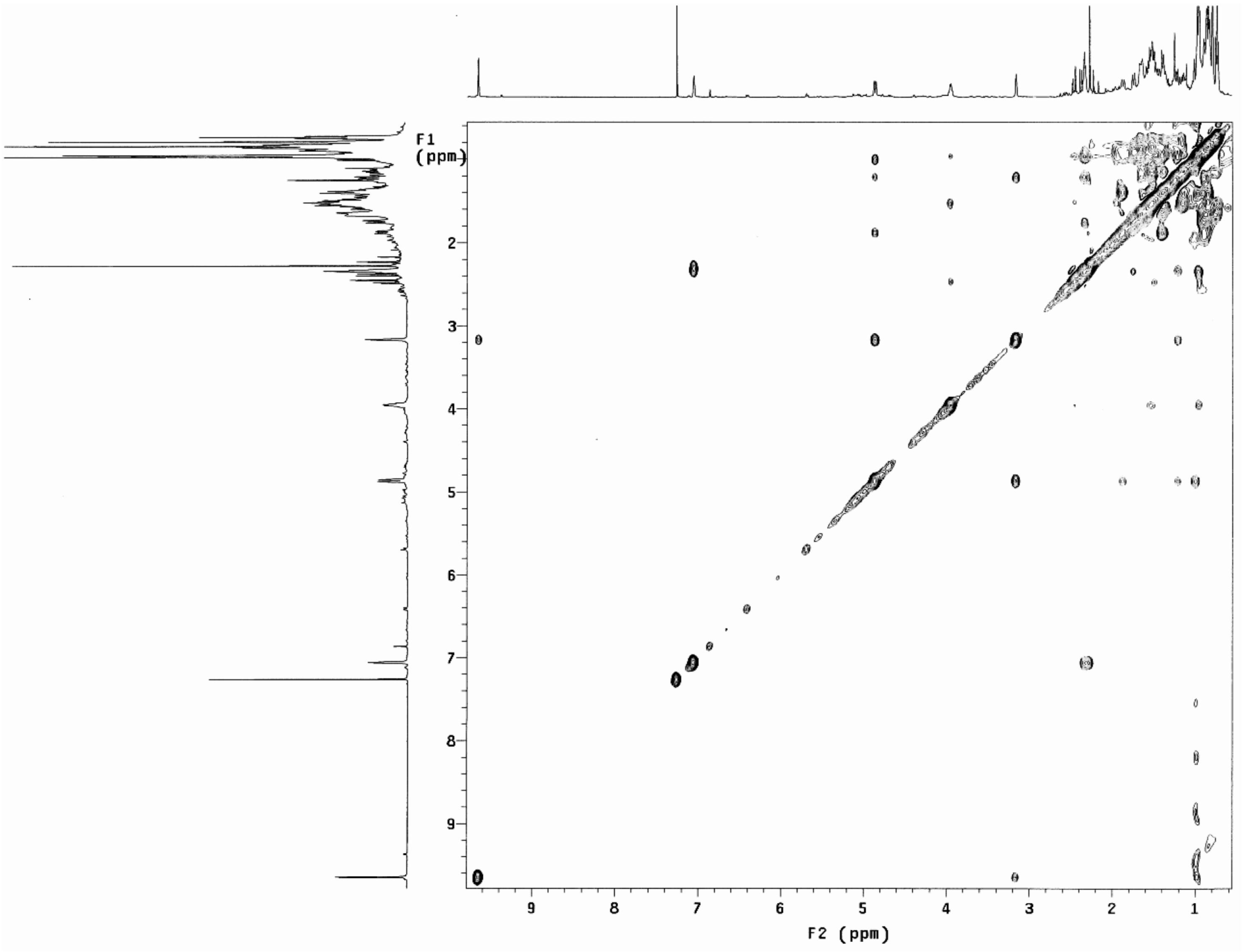

Figure S14: NOESY NMR (500 MHz, $CDCl_3$) spectrum of **2**.

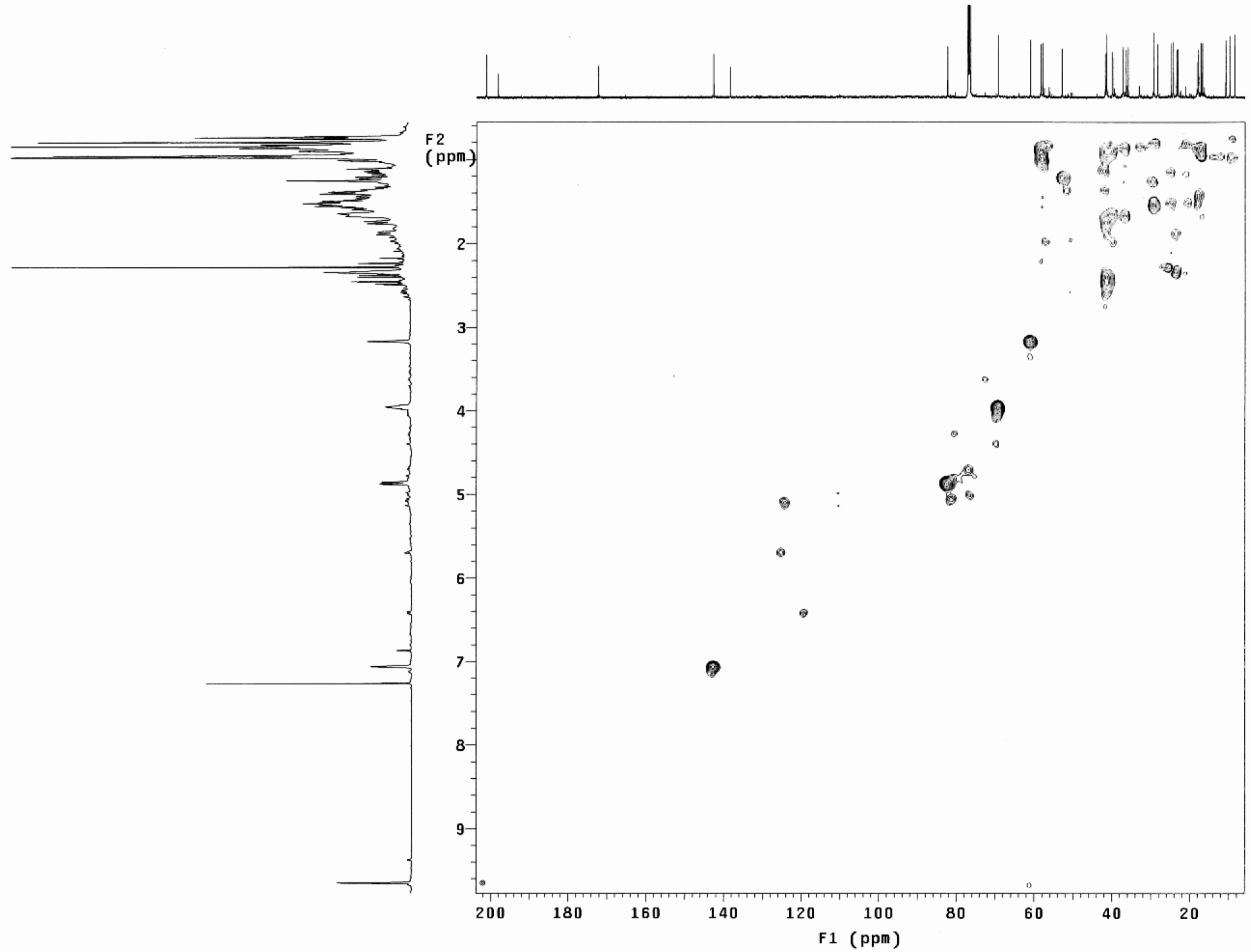

Figure S15: HSQC NMR (500 MHz, $CDCl_3$) spectrum of **2**.

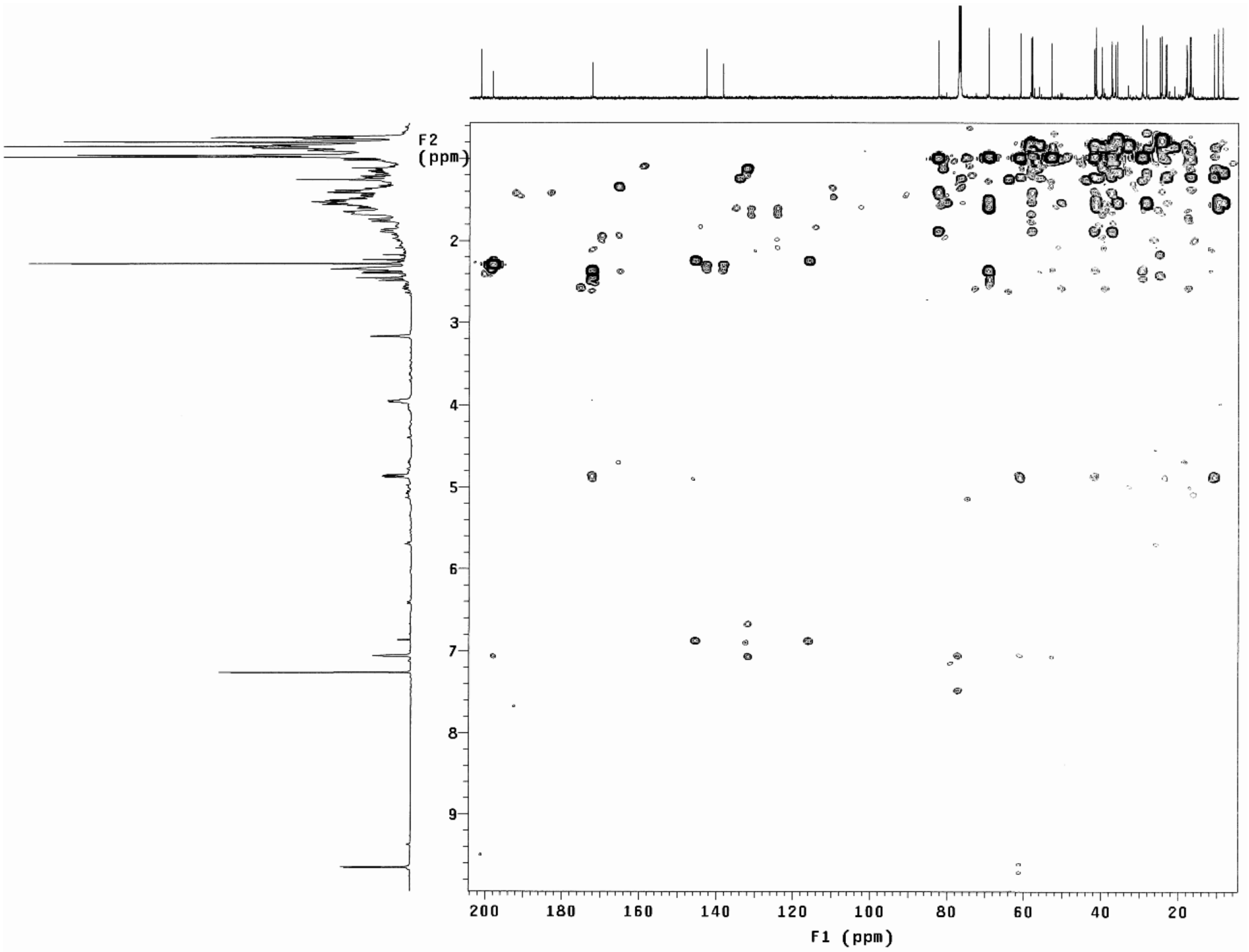

Figure S16: HMBC NMR (500 MHz, $CDCl_3$) spectrum of **2**.

Mass Spectrum SmartFormula Report

Analysis Info

Analysis Name D:\Data\b2\lysp9p_000001.d

Method broadband first signal

Sample Name Lysp-9p

Comment ESI Positive

12/20/2013 10:40:21 AM

Instrument: FT-MS solariX

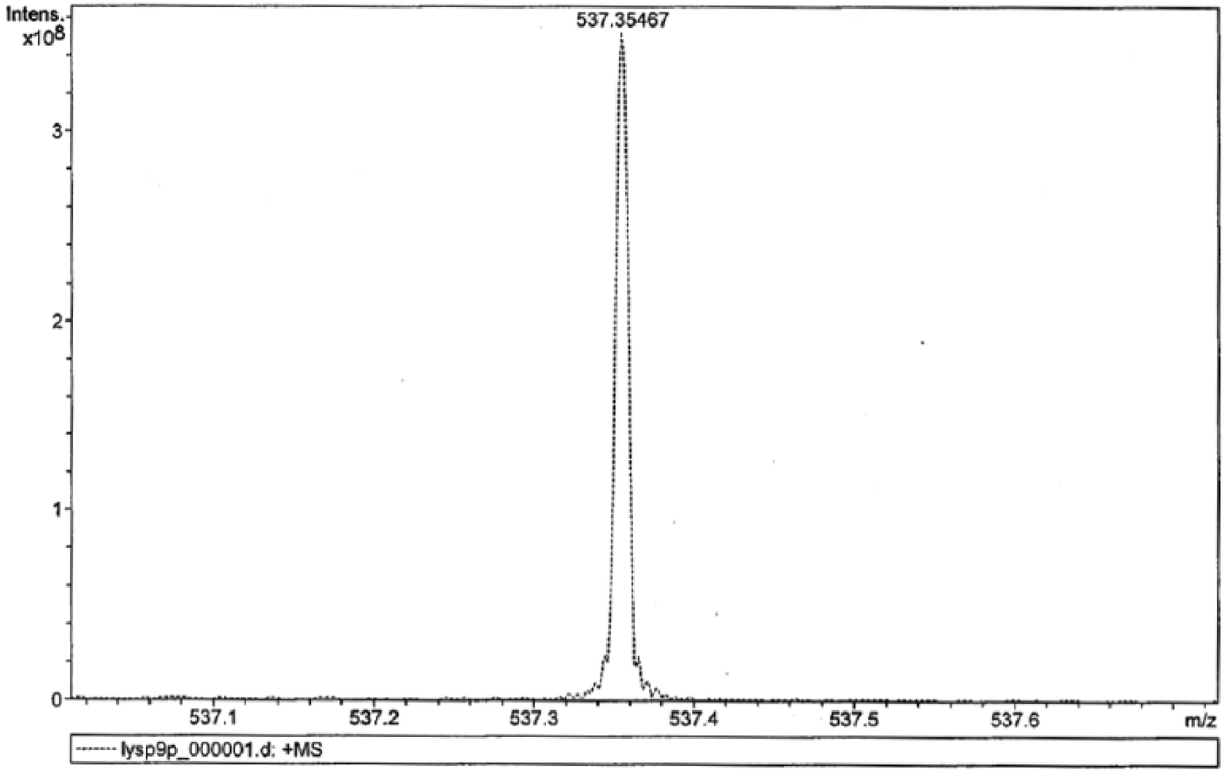

Meas. m/z	#	Formula	Score	m/z	err [mDa]	err [ppm]	mSigma	rdb	e⁻ Conf	N-Rule
537.35467	1	C 32 H 50 Na O 5	100.00	537.35505	0.37	0.70	4.8	7.5	even	ok

Figure S17: HRESIMS spectrum of **2**.

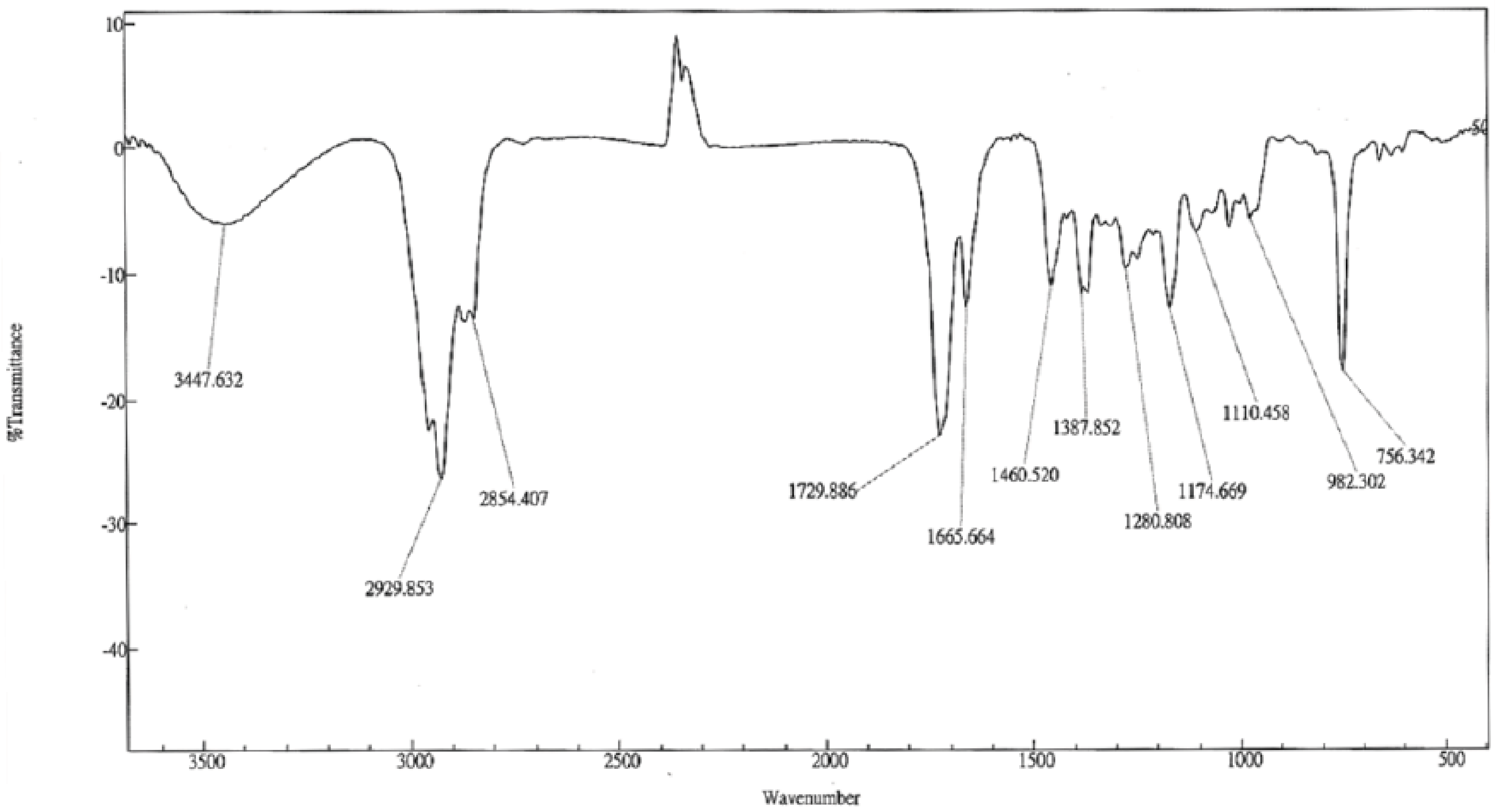

Figure S18: IR spectrum of **2**.

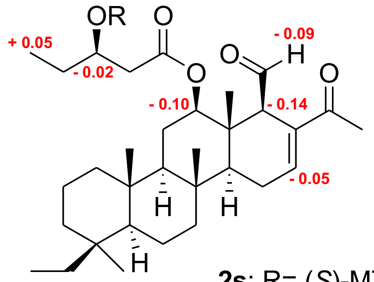

2s: R= (*S*)-MTPA
2r: R= (*R*)-MTPA

Figure S19: Values of $\Delta\delta_{S\text{-}R}$ of the MTPA esters of **2**.

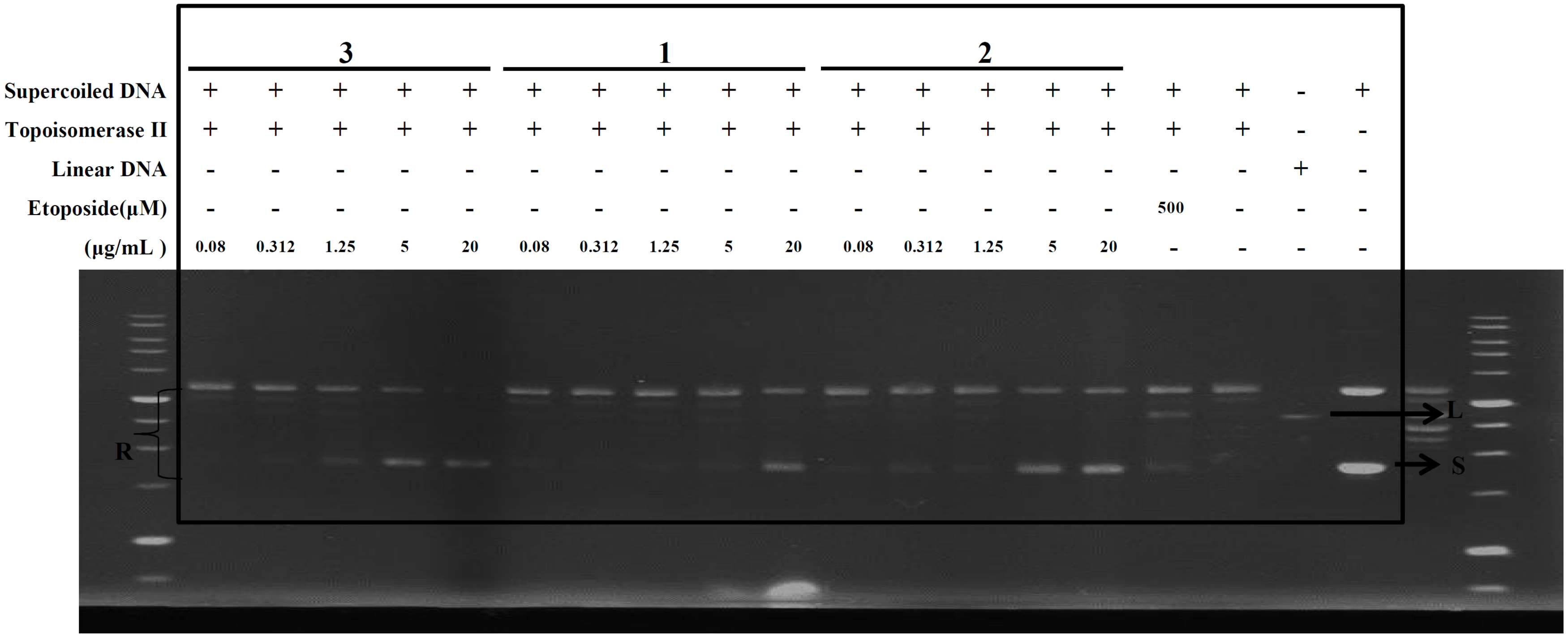

Figure S20: The full length gel of Topo IIα activity.

Effect of compounds **1-3** on topo II activity. Lanes 1-5: **3** (0.08, 0.3125, 1.25, 5, and 20 μg/mL); Lanes 6-10: **1** (0.08, 0.3125, 1.25, 5, and 20 μg/mL); Lanes 11-15: **2** (0.08, 0.3125, 1.25, 5, and 20 μg/mL); Lane 16: positive control, etoposide (500 μM), as topo II poison (induction of linear DNA); Lane 17: plasmid DNA + topo II + solvent control (induction of DNA relaxation); Lane 18: Linear DNA; Lane 19: negative control plasmid DNA (supercoiled DNA)

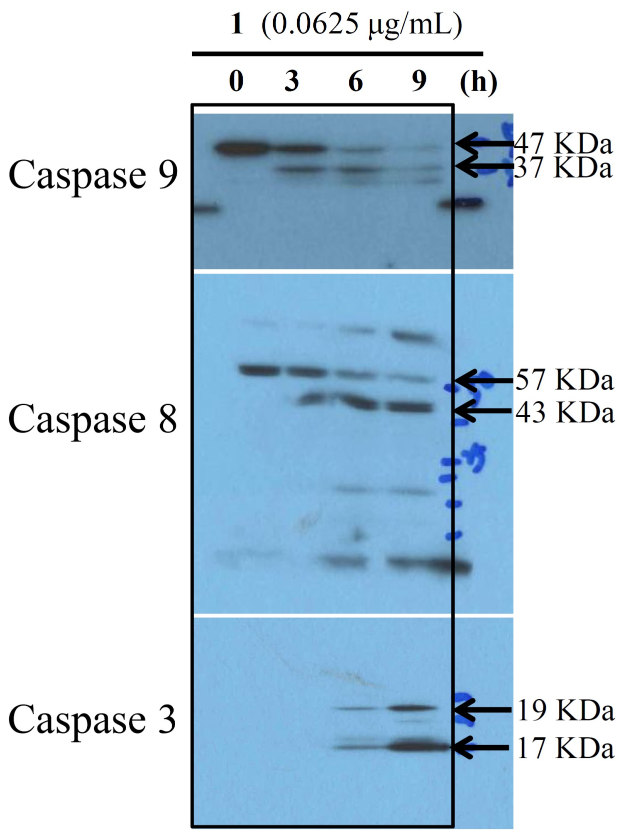

Figure S21: The full length blots of caspase 9, 8 and 3 expressions.

Molt 4 cells were treated with 0.0625 μg/mL of compound **1** for the indicated time. Western blot analysis of Caspase 9 at 47 and 37 KDa, Caspase 8 at 57 and 43 KDa, as well as Caspase 3 at 19 and 17 KDa.
